# Hierarchical deconstruction of mouse olfactory sensory neurons: from whole mucosa to single-cell RNA-seq

**DOI:** 10.1038/srep18178

**Published:** 2015-12-16

**Authors:** Luis R. Saraiva, Ximena Ibarra-Soria, Mona Khan, Masayo Omura, Antonio Scialdone, Peter Mombaerts, John C. Marioni, Darren W. Logan

**Affiliations:** 1 Wellcome Trust Sanger Institute, Wellcome Genome Campus, Hinxton-Cambridge, CB10 1SA, United Kingdom; 2 European Bioinformatics Institute (EMBL-EBI), European Molecular Biology Laboratory, Wellcome Genome Campus, Hinxton-Cambridge, CB10 1SD, United Kingdom; 3 Department of Experimental Genetics, Sidra Medical & Research Center, Qatar Foundation, PO Box 26999, Doha, Qatar; 4 Max Planck Research Unit for Neurogenetics, Max-von-Laue-Strasse 4, 60438 Frankfurt, Germany; 5 Monell Chemical Senses Center, 3500 Market Street, Philadelphia, PA 19104, USA

## Abstract

The mouse olfactory mucosa is a complex chemosensory tissue composed of multiple cell types, neuronal and non-neuronal. We have here applied RNA-seq hierarchically, in three steps of decreasing cellular heterogeneity: starting with crude tissue samples dissected from the nose, proceeding to flow-cytometrically sorted pools of mature olfactory sensory neurons (OSNs), and finally arriving at single mature OSNs. We show that 98.9% of intact olfactory receptor (OR) genes are expressed in mature OSNs. We uncover a hitherto unknown bipartition among mature OSNs. We find that 19 of 21 single mature OSNs each express a single intact OR gene abundantly, consistent with the one neuron-one receptor rule. For the 9 single OSNs where the two alleles of the abundantly expressed OR gene exhibit single-nucleotide polymorphisms, we demonstrate that monoallelic expression of the abundantly expressed OR gene is extremely tight. The remaining two single mature OSNs lack OR gene expression but express *Trpc2* and *Gucy1b2*. We establish these two cells as a neuronal cell type that is fundamentally distinct from canonical, OR-expressing OSNs and that is defined by the differential, higher expression of 55 genes. We propose this tiered experimental approach as a paradigm to unravel gene expression in other cellularly heterogeneous systems.

The olfactory mucosa in the nasal cavity of the mouse is a complex and heterogeneous tissue composed of neuronal and non-neuronal cell types. The main olfactory epithelium (MOE) component of the olfactory mucosa contains mature and immature olfactory sensory neurons (OSNs), horizontal basal cells (HBCs), globose basal cells (GBCs), and sustentacular cells (SUSs). The submucosa below the MOE harbors olfactory ensheathing cells (OECs), blood and lymph vessels, and glandular and cavernous tissues^1^,^2^. Odorant reception occurs primarily in the MOE, via >1,000 G-protein coupled olfactory receptors (ORs)^3^. Each mature OSN is thought to express a single intact OR gene in a monoallelic fashion^4^,^5^. ORs signal in a combinatorial fashion to maximize odorant detection and discrimination^4^,^6^. OSNs expressing the same OR gene are scattered within a characteristic region of the MOE, and their axons coalesce into a few glomeruli in the main olfactory bulb where they synapse with second-order neurons in the olfactory pathway^7^,^8^,^9^. In turn these second-order neurons transmit signals to the olfactory cortex and other regions of the brain. A population of OSNs defined by the expressed OR gene constitutes the elementary unit of olfactory sensory input to the brain^10^.

We have recently characterized the transcriptome of C57BL/6 mouse olfactory mucosa by deep RNA sequencing (RNA-seq), and generated a comprehensive gene expression profile for this tissue^11^. But this approach averages each gene’s expression level across the many different cell types that are present in these crude tissue scrapes, thus obscuring the heterogeneity of cell types and subtypes. Moreover, genes encoding proteins that are abundantly secreted from olfactory glands dominate this transcriptome, to the extent that genes expressed in small subsets of cells, like most OR genes, are significantly underrepresented^11^. Even within one MOE cell type - canonical, OR-expressing OSNs - the molecular heterogeneity is substantial: there are >1,000 distinct OSN subsets, each defined by the expressed OR gene^11^. Multiple additional types of chemosensory neuron have been identified in the mouse MOE, including cells expressing the guanylyl cyclase D receptor (*Gucy2d* or GC-D)^12^ and cells expressing trace-amine associated receptors (TAARs)^13^. These smaller cell populations have been the focus of some recent functional studies^14^, but the full molecular identity and the extent of heterogeneity among the vast majority of chemosensory neuronal cell types in the MOE remain unknown.

Here we combine RNA-seq with Fluorescence Activated Cell Sorting (FACS) in a hierarchical fashion: from crude tissue samples containing MOE down to single mature OSNs. Our three-step approach is based on purification of mature, GFP-expressing OSNs from whole olfactory mucosa (WOM) scrapes of heterozygous OMP-GFP mice^15^. Olfactory marker protein (OMP) is a widely accepted marker for mature OSNs, but some chemosensory neurons in the nasal cavity such as *Gucy2d*-expressing neurons express little or no OMP^16^. We find evidence for expression of 1,087 from 1,099 (98.9%) intact OR genes within these FACS-sorted cell populations, with intact referring to a full length (~1 kb), uninterrupted open reading frame (ORF). Importantly, the expression levels in sorted OSNs are proportional to their levels in WOM samples, indicating that our methods of tissue dissociation and FACS reliably retrieve and isolate the many distinct OSN subsets from the MOE component of WOM samples in a highly representative manner. We uncover a hitherto unknown bipartition between OMP-GFP^low^ and OMP-GFP^high^ expressing OSNs, representing a discrete subdivision within the OMP-positive OSN population defined by markers of neuronal maturity. By RNA-seq of 21 single OMP-GFP^high^ expressing OSNs, we find that 19 cells express a single intact OR gene abundantly, with some evidence of much lower levels of expression for several other OR genes. We demonstrate that the monoallelic expression of an OR gene in a single OSN is extremely tight in the 9 single OSNs for which single nucleotide polymorphisms (SNPs) enable discrimination between the two alleles in the mixed 129P2 x C57BL/6 background of the OMP-GFP strain. Finally, with the remaining two OMP-GFP^high^ cells, we molecularly characterize a recently described type of chemosensory neuron in the MOE: type B Trpc2^+^ cells^17^,^18^,^19^. We demonstrate that these two cells do not express ORs, TAARs, *Gucy2d* or any of the other known chemosensory G-protein coupled receptors. We identify 55 upregulated genes that establish these cells as a novel neuronal type within the MOE, which is fundamentally distinct from canonical OSNs.

## Results

### The transcriptional profile of mature olfactory sensory neurons

To characterize gene expression in mature OSNs, we need to purify them away from the many other cell types that are present within the crude tissue samples that can be scraped from the nasal cavity and contain not only pure MOE but also submucosa and adjacent tissues.

**Figure 1:**
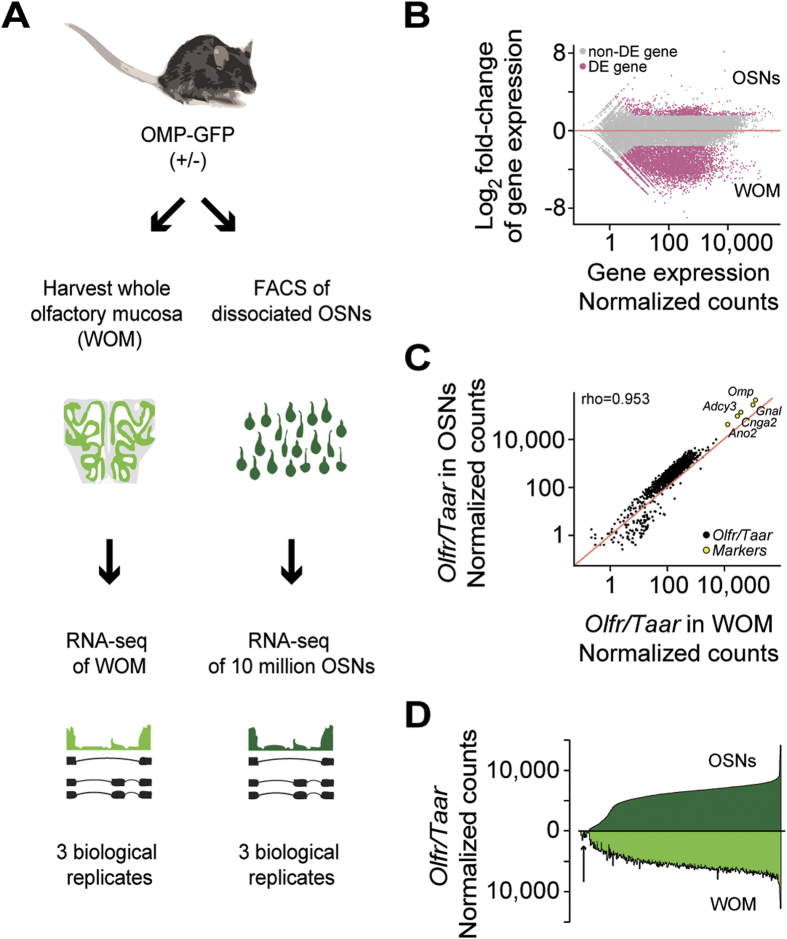
Differential expression analysis of mouse olfactory sensory neurons (OSNs) and whole olfactory mucosa (WOM). (**A**) Schematic of the RNA-seq experimental strategy. After dissection of the WOM of OMP-GFP (+/−) male and female mice, pools of ~10 million OSNs were collected by FACS. RNA was extracted from these, along with WOM samples, cDNA generated, and libraries were amplified for deep sequencing. (**B**) Differential gene expression analysis between the transcriptomes of OSNs and WOM. Statistically significant differentially expressed genes (fold-change >3; FDR < 5%) are highlighted in pink. (**C**) Comparison of OR and TAAR gene expression levels. A scatter plot of OR and TAAR gene expression levels (black) in the WOM versus the sorted OSNs reveals a strong correlation. The red line represents the 1:1 diagonal. Classical marker genes for mature OSNs (yellow) are similarly enriched. (**D**) Distribution of OR and TAAR gene expression (normalized counts) as a barplot, for both OSNs and WOM. Genes are displayed in ascending order of their expression values in OSNs (dark green). Matching values are plotted for WOM (bright green), demonstrating that the dynamic distribution of OR gene expression is largely conserved, but differs in OR genes expressed at low levels in WOM (arrow).

We FACS-sorted cell suspensions of dissociated WOM samples from 25-day old, heterozygous gene-targeted mice engineered to express green fluorescent protein (GFP) from the endogenous Olfactory Marker Protein (OMP) promoter^15^ (Fig. 1A and Supplementary Fig. S1A). We applied RNA-seq to three independent pools of ~10 million OMP-GFP^+^ OSNs (hereafter referred to as “OSNs”), which is approximately the number of OSNs present in the nose of a single adult mouse^20^, and to three WOM samples from mice of the same age, strain, and mixed genetic background (Fig. 1A). We find that gene expression levels are highly correlated between independent biological replicates of WOM (Spearman’s rho = 0.975) and between OSN pools (Spearman’s rho = 0.969) (Supplementary Fig. S1B). A differential expression (DE) analysis identified 790 genes that are expressed higher in OSNs relative to WOM (fold-change > 3; FDR < 5%) (Fig. 1B), 50.1% of which are OR or TAAR genes (Supplementary Data S1). A gene ontology (GO) analysis revealed that genes more highly expressed in the OSN pools relative to WOM are significantly enriched in terms related to the olfactory transduction pathway, as well as in G-protein coupled amine receptor activity (Supplementary Data S1). Other enriched GO terms include genes related to synaptic vesicles, branching morphogenesis of a nerve, and peptide hormone processing. Of the 5,227 genes that are expressed higher in WOM (fold-change <0.33; FDR < 5%), 55.46% are expressed at least ten times higher than in the OSN pools, suggesting these are likely to be restricted to entirely different cell types within the WOM samples. To validate these observations, we interrogated an existing microarray dataset of OSN gene expression from the same strain of OMP-GFP mice^21^. We find that genes enriched in our FACS-sorted OSNs are consistent with OMP^+^ enrichment in Sammeta *et al.*, and conversely, that genes enriched in our WOM samples are consistent with OMP^–^ enrichment in the same study^21^ (Supplementary Fig. S1C). We surveyed the top 200 DE genes between OSNs versus WOM to identify novel genes likely to be involved in olfaction (Supplementary Fig. S1D). Among the most abundant genes that are more highly expressed in OSNs are *Gnal*, *Gnb1*, *Adcy3,* and *Cnga2*, all of which are involved in canonical odorant-mediated signal transduction. We identified a number of novel genes of interest because of their high differential expression in OSNs, such as *Fstl5* and *Cmip* (the 8^th^ and 20^th^ most abundant DE genes in OSNs respectively, Supplementary Data S1).

We next compared OR and TAAR gene expression levels between OSNs and WOM. To capture the full abundance levels, we mapped the sequencing data to full-length transcripts^11^ instead of the much shorter Ensembl transcript models, which typically only capture the coding region (Supplementary Fig. S1E). We find that OR and TAAR gene expression levels are strongly correlated between the OSN and the WOM samples (Spearman’s rho = 0.953) (Fig. 1C,D). Indeed only 19 (1.52%) OR genes, all expressed at very low levels in the WOM, lack representation in any of the OSN pools, and 13 of these are annotated as pseudogenes (Fig. 1D). On average, OR and TAAR genes, as well as other OSN-specific markers, are expressed 2.56 fold higher in the OSN samples than in the WOM (Fig. 1C), consistent with mature OSNs comprising a minority of the cells within the WOM.

Taken together, we have generated the transcriptome of almost the entire mouse OSN repertoire by RNA-seq, identifying hundreds of genes that are OSN-specific and thousands that are restricted to other cells contained within the WOM. Our exhaustive sampling approach maintains excellent proportionality of abundance between the purified OSNs and the WOM. There are thus no major differences in recovery of OSN subsets expressing the various OR genes by tissue dissociation and FACS-sorting compared to their *in situ* representation within the MOE.

### A bipartition of mature olfactory sensory neurons

While performing FACS of OSNs on heterozygous OMP-GFP mice, we noticed two distinct subpopulations based on GFP intensity (Supplementary Fig. S1A). As OMP is widely accepted as a marker of mature OSNs^22^,^23^, these may represent subpopulations of mature neurons at distinct stages of maturation.

**Figure 2:**
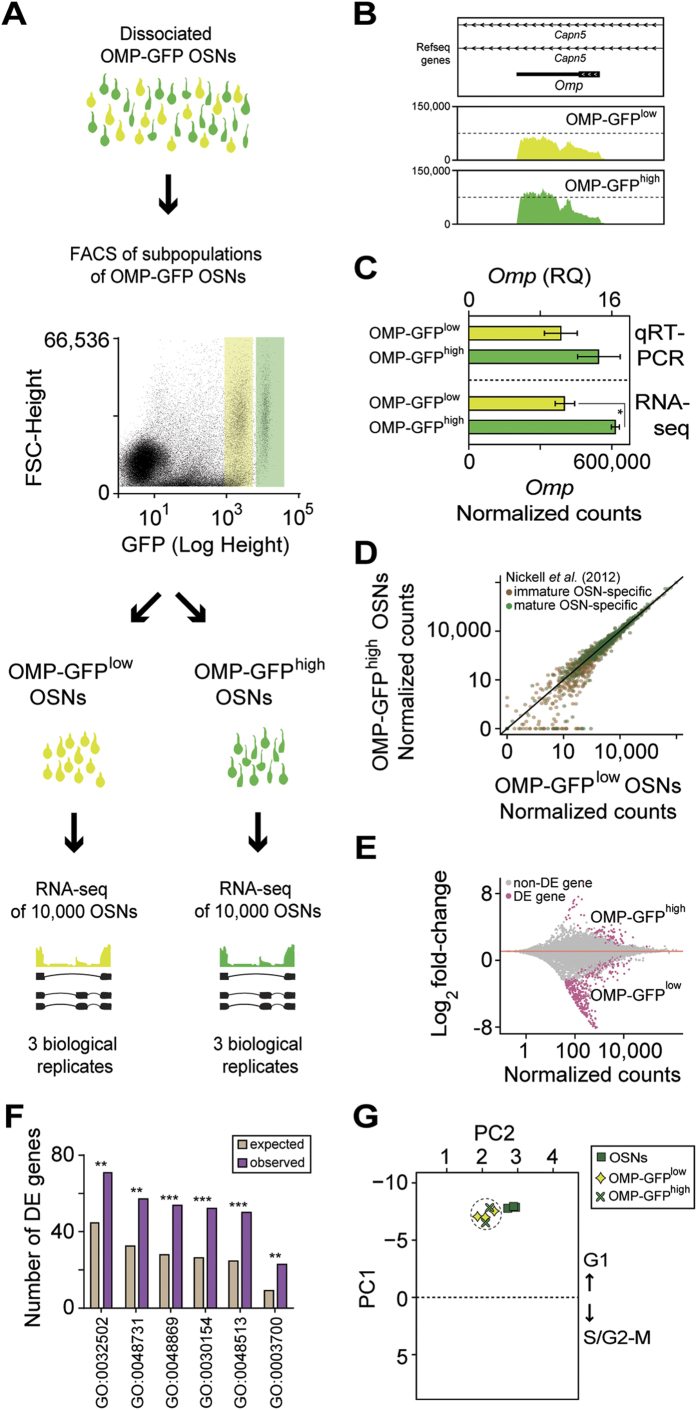
Differential expression analysis of mature OSN subpopulations. (**A**) Schematic of the RNA-seq experimental strategy. The WOM of OMP-GFP (+/−) male mice was dissected and pools of ~10,000 OSNs from two non-overlapping subpopulations, OMP-GFP^low^ (light green) and OMP-GFP^high^ (dark green), were collected by FACS gating based on fluorescence intensity. Subsequently the RNA was extracted, cDNA generated, and libraries were amplified for deep-sequencing. (**B**) Representative coverage plot showing the distribution of RNA-seq reads across the *Omp* locus (which is located within an intron of *Capn5*) is similar in both subpopulations. (**C**) The number of *Omp* normalized counts is 1.5 times higher in the OMP-GFP^high^ population (unpaired *t*-test, 2 tails, *P = 0.025). Analysis of the same samples by TaqMan qRT-PCR verified this increase. (**D**) Scatter plot showing the expression levels of previously reported immature (brown) and mature (green) OSN-specific markers^24^ in the OMP-GFP^low^ and OMP-GFP^high^ OSNs. Both sets of genes are expressed at equivalent levels in both populations (Wilcoxon rank sum test, P = 0.45 and P = 0.22 for mature and immature gene sets), but mature specific genes are expressed considerably higher (Wilcoxon rank sum test, P < 2.2–16 and P < 2.2–16 for OMP-GFP^low^ and OMP-GFP^high^). (**E**) Differential gene expression analysis between the transcriptomes of OMP-GFP^low^ and OMP-GFP^high^ OSNs. Statistically significant differentially expressed (FDR < 5%) genes are highlighted in pink. (**F**) Functional terms enrichment analysis between the OMP-GFP^low^ and OMP-GFP^high^ differentially expressed genes identified a total of 88 GO terms significantly overrepresented (FDR < 5%). The bar graph indicates the expected and observed number of DE genes for six of these GO terms (*P ≤ 0.05, **P ≤ 0.01, ***P ≤ 0.001). The full list and GO category names is in Supplementary Data S2. (**G**) Projection of the OSNs, OMP-GFP^low^ and OMP-GFP^high^ samples onto the cell cycle Principal Component Analysis (PCA) that differentiates between cell cycle stages (see Supplementary Fig. S2B and Supplementary Methods for details). Principal Component 1 (PC1) segregates the samples based on their cell cycle stage, with samples in G1 having negative values and samples in S/G2-M positive values. All tested samples are in the G1 stage.

To investigate this hypothesis, we performed RNA-seq on three pools of 10,000 OSNs sampled from the two subpopulations (termed OMP-GFP^low^ and OMP-GFP^high^), each time from a single heterozygous OMP-GFP mouse (Fig. 2A). We find that gene expression levels are highly correlated between biological replicates among the OMP-GFP^low^ pools and among the OMP-GFP^high^ pools (Spearman’s rho = 0.89–0.91) (Supplementary Fig. S2A). One OMP-GFP^high^ sample yielded a substantially lower number of sequencing reads (Supplementary Data S2) so we excluded it from our subsequent analyses. Consistent with the FACS distributions, we confirmed ~1.5 higher levels of *Omp* expression in the OMP-GFP^high^ compared to the OMP-GFP^low^ subpopulations, independently by qRT-PCR and RNA-seq (Fig. 2B,C).

We next investigated the expression levels of 1,235 genes that have been reported to be differentially enriched in immature (670) or mature (565) OSNs^24^. We find that the mature OSN-enriched genes are robustly expressed at similar levels in both the OMP-GFP^low^ and OMP-GFP^high^ subpopulations (Wilcoxon rank sum test, P = 0.45) (Fig. 2D). Moreover, the mature OSN-specific genes are expressed at significantly higher levels than the immature OSN-specific genes in both populations (Wilcoxon rank sum test, P < 2.2–16 for both OMP-GFP^low^ and OMP-GFP^high^), suggesting that OMP-GFP^low^ and OMP-GFP^high^ cells both correspond to mature OSNs. Further, the expression of genes recently implicated in establishing and maintaining monogenic expression in OSNs^25^,^26^ do not significantly differ between the subpopulations (Supplementary Fig. S2B). Among genes involved in OSN axon guidance^27^, only *Robo2* has a small but significant decrease in expression in the OMP-GFP^high^ subpopulation (Supplementary Fig. S2C).

Consequently, we carried out an unbiased comparison between the two subpopulations to determine how they differ in their transcriptional profile. We identified 537 DE genes (FDR < 5%) of which 420 (78.2%) are more highly expressed in the OMP-GFP^low^ subpopulation (Fig. 2E). Applying GO term analysis to these genes in order to obtain indications about the cellular processes they regulate, we find a statistically significant enrichment for terms related to development, morphogenesis, negative regulation of neuronal differentiation and positive regulation of cell proliferation (Fig. 2F; and Supplementary Data S2).

A hallmark of neuronal differentiation/maturation is the exit from the cell cycle, late in the G1 stage, by the neural progenitors^28^. We investigated the cell-cycle phase of the OSNs from each subpopulation. We selected 971 genes with a “cell cycle” GO annotation, calibrated their expression against a published dataset of staged ES cells (Supplementary Fig. S2B), and analyzed three test sets (blastomeres, brain, and liver, Supplementary Fig. S2C) to ensure that we can accurately allocate RNA-seq data to different cell cycle stages (see Supplementary Methods). Testing the RNA-seq datasets from ~10 million OSNs, OMP-GFP^low^ and OMP-GFP^high^ cells, we found that all samples are allocated to the G1 phase (Fig. 2G).

We have thus uncovered a hitherto unknown bipartition among mature OSNs. Both subpopulations are postmitotic, but the OMP-GFP^low^ subpopulation, while mature based on its OMP expression level and expression profile of known marker genes, appears to be somewhat less mature than the OMP-GFP^high^ subpopulation.

### RNA-seq of single olfactory sensory neurons

Having identified and isolated a subpopulation of OSNs that represent the most mature state (OMP-GFP^high^), we proceeded to the ultimate objective of this study: characterizing gene expression in single mature OSNs by single-cell RNA-seq.

**Figure 3:**
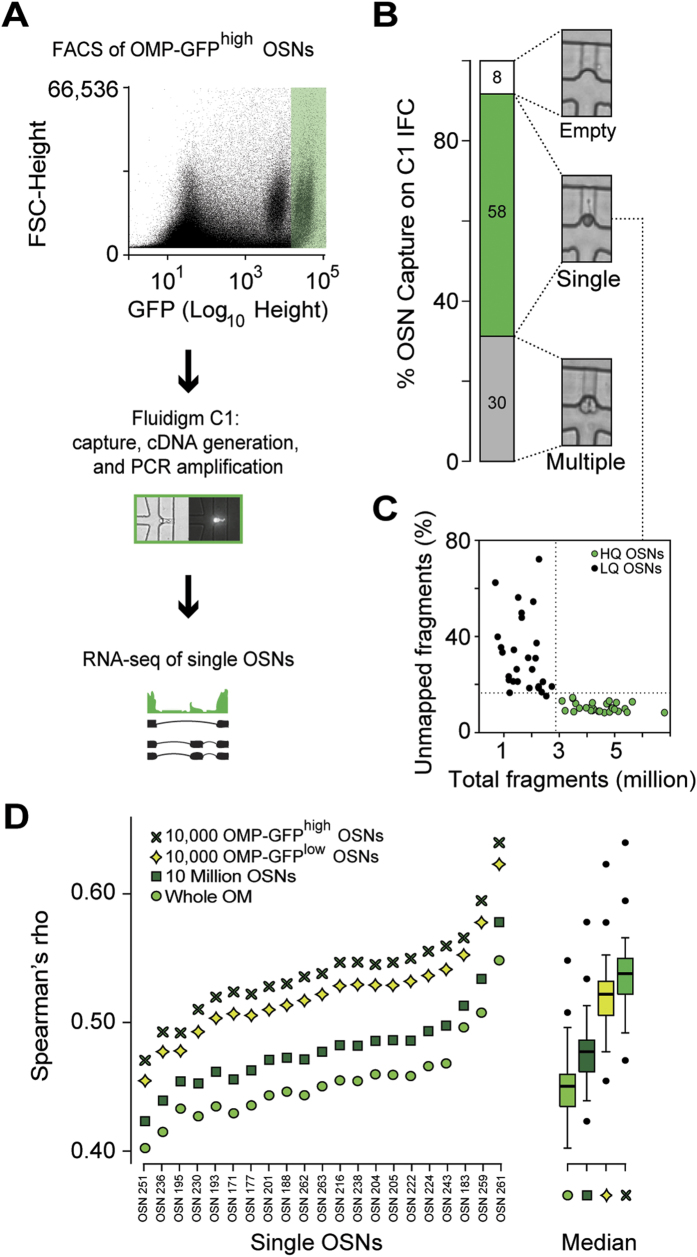
RNA-seq in single mature OSNs. (**A**) Schematic of the single-cell RNA-seq experimental strategy. After dissection of WOM of a heterozygous OMP-GFP mouse, 5,000 mature OMP-GFP^high^ OSNs were collected by FACS into a C1 IFC (10–17 μm, Fluidigm). Using the Fluidigm C1 platform, single cells were sorted, imaged, RNA extracted, cDNA generated, and libraries amplified for deep-sequencing. A representative bright field and fluorescence image for a captured single OSN is shown in the middle panel. (**B**) Capture rate for single cells: 30 wells captured more than one cell and/or debris, 58 captured single cells, and 8 were empty. Representative bright field images are shown for each of these categories. (**C**) The 58 captured single OSNs were subjected to stringent, hierarchical quality control criteria, which defined low-quality (LQ, black dots) and high-quality cells (HQ, green dots, see also Supplementary Fig. S3; and Supplementary Methods). (**D**) Spearman correlation coefficients of the 21 single cells used in our downstream analysis with the WOM and OSN populations (10 million, OMP-GFP^low^ and OMP-GFP^high^ OSNs). As expected, the single cells correlate best with the OMP-GFP^high^ OSNs, followed by the OMP-GFP^low^ OSNs, the 10 million OSNs and finally WOM. To the right of the graph, boxplots show the cumulative data for each comparison. The thick black horizontal bar corresponds to the median and data points that are outside 1.5 times the interquartile range (the box) are indicated as outliers (black dots).

We used a Fluidigm C1 microfluidic system to capture 58 single cells from the OMP-GFP^high^ subpopulation of an individual mouse (Fig. 3A). The remaining wells in the 96-well capture chip were either empty, contained visible debris and/or more than one cell (Fig. 3B). By applying stringent, hierarchical quality control criteria to the RNA-seq data, we further excluded 37 cells. We focused our downstream analysis on the remaining 21 cells (Fig. 3C; Supplementary Fig. S3. See Methods and Supplementary Methods for details of the quality control procedure). On average 4.4 million sequence fragments were obtained from a single cell. Each fragment provides ~200 bp of cDNA sequence, which we mapped to the mouse reference genome (Supplementary Data S3). Based on their expression of cell cycle genes, all 21 cells were allocated to the G1 stage, consistent with mature neurons (Supplementary Fig. S3C). To verify that these 21 cells belong to the OMP-GFP^high^ OSN subpopulation, we compared their individual transcriptomes to the gene expression profiles derived from WOM, the pools of 10 million OSNs and the bulk OMP-GFP^low^ and OMP-GFP^high^ OSN subpopulations. As expected, all 21 cells correlate better with the OSN samples than with the WOM samples (paired t-test, P < 2.2-16), and better with the OMP-GFP^high^ than the OMP-GFP^low^ OSN samples (paired t-test, P < 2.2-16) (Fig. 3D). We detect, on average, 4,717 +/− 175 (SEM) genes per single cell. Collectively, 13,582 different genes are expressed in at least one cell, representing 74.2% of the genes expressed in the OMP-GFP^high^ OSN pools.

**Figure 4:**
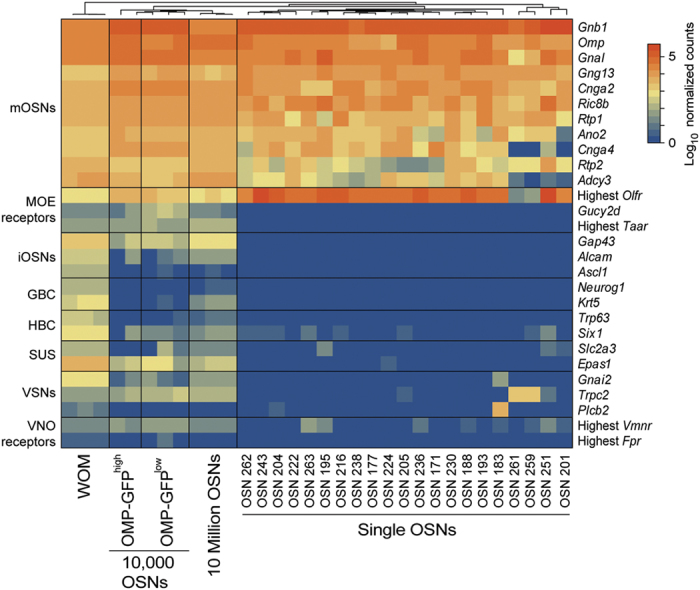
Captured single cells express canonical marker genes of mature OSNs. Heatmap of the expression of canonical olfactory cell-specific markers across the WOM and all OSN samples analyzed. While the WOM and OSN population samples express markers for all cell types, the single cells specifically express high levels of mature OSN markers. mOSNs: mature OSNs, iOSNs: immature OSNs, GBCs: globose basal cells, HBCs: horizontal basal cells, SUSs: sustentacular cells, VSNs: vomeronasal sensory neurons.

Next we examined expression of a catalog of genes that are widely accepted to characterize distinct cell types within the MOE and the vomeronasal organ (VNO). Satisfyingly, we find that all 21 cells show high expression of genes characteristic of mature OSNs (such as *Omp*, *Gnal*, *Cnga2*, *Ano2*, and *Adcy3*), and low or no expression of markers of other MOE cell types (such as *Gucy2d* and *Taar*), of immature OSNs (such as *Gap43*, *Ascl1*, and *Neurog1*), and of vomeronasal sensory neurons (such as *Vmnr* and *Fpr* genes) (Fig. 4). We assessed the expression of 87 additional marker genes that have been identified from microarray and other large-scale analyses of olfactory and vomeronasal mucosae, but have been less well characterized^21^,^24^,^29^,^30^,^31^. Again, we observe a strong enrichment for expression of marker genes from mature OSNs (Supplementary Fig. S4).

Taken together, we have captured and extracted high-quality mRNA and sequenced the corresponding cDNA from 21 OSNs. By preselecting OMP-GFP^high^ OSNs, we ensured that the captured cells were sampled only from the most mature OSN subpopulation, a distinction that cannot be made when picking single cells under a microscope. Our single-cell RNA-seq analyses confirm the source and identity of all 21 cells as mature OSNs, with no contamination by other MOE or VNO cell types.

### Abundant and monogenic OR gene expression

Mature OSNs are functionally distinguished by the OR gene they express^4^,^6^,^10^. They are thought to adhere to the one neuron – one receptor rule^32^,^33^ and to exhibit monoallelic expression of the expressed OR gene^5^.

**Figure 5:**
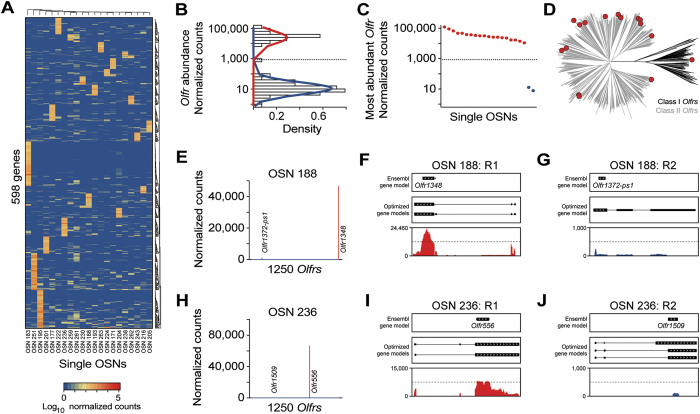
OR gene expression patterns single OSNs. (**A**) Heatmap of the expression levels of the most variable genes in single OSNs. All 598 highly expressed genes (≥1000 normalized counts in at least 1 sample) with a coefficient of variation ≥4 are represented. The identity of these genes are presented in Supplementary Data S3. (**B**) Normal-like distributions were fitted for all lowly expressed (blue line) and highly expressed (red line) OR genes. Only genes at or above the intersection of both curves (855 normalized counts) were considered as abundantly expressed. (**C**) Expression level of the most abundant OR in each of the 21 single OSNs. The most abundant ORs in OSN 259 and OSN 261 are expressed at extremely low levels. (**D**) Phylogenetic tree of all mouse OR genes (Class I and II in black and grey, respectively). The most abundant ORs in all the single OSNs analyzed are depicted as red circles. (**E**) Expression levels of all ORs in OSN 188. The two most abundant ORs in that cell are indicated. (**F,G**) Coverage plots of the RNA-seq data for OSN 188 mapping to the first (*Olfr1348*, (**F**)) and second (*Olfr1372-ps1*, (**G**)) most abundant ORs. Boxes correspond to exons and arrowheads indicate the strand of the gene. The existing Ensembl annotations are shown in the top box. Optimized OR gene models^11^ used in our analysis are shown in the middle box. The sequencing data mapping to the OR gene models is below. (**H**) Expression levels of all ORs in OSN 236. The two most abundant ORs in that cell are indicated. (**I,J**) Coverage plots of the RNA-seq data for OSN 236 mapping to the first (*Olfr556*, (**I**)) and second (*Olfr1509* (**J**)) most abundant ORs. Boxes correspond to exons and arrowheads indicate the strand of the gene. The existing Ensembl annotations are shown in the top box. Optimized OR gene models^11^ used in our analysis are shown in the middle box. The sequencing data mapping to the OR gene models is below.

We would expect OR genes and possible other regulators of OSN identity to show heterogeneous patterns of expression across the OSN population. We therefore computed the coefficient of variation (CV = standard deviation/mean) for the 4,121 genes with >1000 normalized counts in at least one of the 21 single OSNs. This procedure allowed us to identify 598 genes with highly variable expression patterns across cells (CV > 4, Supplementary Data S3). These genes are distributed relatively evenly across the single OSNs (Fig. 5A), except for one unusually variable cell (OSN 183), which accounted for 89 of the 598 genes (15%). The genes with high CV in this cell are enriched for GO terms related to chemokine receptor binding, cytokine binding and activity, antigen processing and presentation, and regulation of lymphocyte activation, suggesting a stressed cellular state. The remaining 509 genes that are expressed in one or a small number of the other 20 OSNs are statistically enriched only in GO terms related to G-protein coupled receptor (GPCR) signaling and transduction. Importantly, the majority of the high-CV genes within these GO terms are OR genes. There are a few other orphan GPCR genes with a CV > 4: *Gpr32*, *Gpr123, Gpr125* and *Gpr160* (Supplementary Fig. S5A). We carried out a similar analysis to assess whether there is any enrichment for shared protein domains in the predicted amino-acid sequences. As expected, we identified the seven-transmembrane receptor domain that is typical for GPCRs (defined in the protein family database, PFAM, by domain PF00001). Curiously, we also identified a statistical enrichment in 18 genes encoding zinc-finger motifs (PFAM: PF00096) (Supplementary Fig. S5B). Thus, OR genes systematically distinguish individual OSNs. We speculate that the highly restricted expression patterns of several zinc-finger and orphan GPCR genes may reflect lineages or subtypes within the OSN repertoire.

We next investigated the expression of all OR and TAAR genes, including pseudogenes, within the 21 single cells. In total, we found 476 instances where an OR gene had at least one fragment mapped in at least one OSN; in contrast, no fragments mapped to TAAR genes. In 86% of these 476 instances, however, the mapped fragments cover less than one third of the length of the OR gene (Supplementary Fig. S5C), consistent with non-specific transcription and/or mismapped reads (Supplementary Fig. S5D). The remaining 65 OR genes segregate into two distinct classes based on transcript abundance (Fig. 5B). A total of 45 OR genes fall in a low-abundance class with a mean expression of 15.99 ± 2.7 (SEM) normalized counts (Supplementary Fig. S5C,E). The remaining 20 OR genes are abundantly expressed (mean 36,162.46 ± 6,238.7 SEM normalized counts) and have reads densely mapped across the majority of the full transcript (Supplementary Fig. S5C,F).

We then analyzed the OR genes expressed in each individual OSN, using the intersection between these two distributions at 855 normalized counts (Fig. 5B; and Supplementary Fig. S5C) to define whether an OR gene is of high or low abundance. We find that 19 of the 21 OSNs express a single intact OR gene at extremely abundant levels (above the intersection, Fig. 5B,C). On average the OR genes rank as the 6^th^ most abundantly expressed genes in the cells (range 1 to 15), when measured by normalized counts, with only *Stoml3*, *Gnb1*, *Malat1* and *Calm1* being consistently expressed more strongly (Supplementary Data S3). Consistent with the ~1:10 ratio of class I to class II OR genes in the mouse genome^34^, all but one (*Olfr556*) of these abundantly expressed OR genes encode a class II OR protein. Moreover, the set of OR genes expressed are distributed throughout the OR phylogeny (Fig. 5D). Thus, the 19 OR-expressing mature OSNs that we captured appear broadly representative of the full OR gene repertoire.

In OSN 188, we observed that O*lfr1372-ps1*, an OR pseudogene, is coexpressed at moderate levels (exactly at the intersection of 855 normalized counts, between the low/high abundance distributions) alongside an intact OR gene (*Olfr1348*, Fig. 5E–G). We reconstructed the O*lfr1372-ps1* transcript from the mapped reads and confirmed that it has a truncated ORF. The coexpression of two OR genes (*Olfr1348* and O*lfr1372-ps1*) in OSN 188 is consistent with evidence that OR genes with an experimentally interrupted ORF can still be expressed, but lose their monogenic character^35^. The 19 OSNs also have between 11 and 28 additional OR genes with evidence of expression, but all with extremely low normalized counts. Indeed, after excluding pseudogenes, the most highly expressed OR gene is on average over 1,000 times more abundant than the next highest OR gene expressed (Fig. 5H–J; and Supplementary Fig. S5G), and we find no evidence of phylogenetic or chromosomal proximity between the two (Supplementary Data S3).

In two cases, we identified a pair of cells that abundantly express the same OR gene: OSN 171 and OSN 177 each express *Olfr728*, and OSN 222 and OSN 263 each express *Olfr55*. Interestingly, the additional OR genes with very low normalized counts are not shared between the cells of each pair (Supplementary Fig. S5G). Their expression is thus not coordinated with the choice of abundant OR gene, nor are their low expression counts a consequence of mismapping of a fraction of the reads of the abundantly expressed OR gene. To assess whether this pattern of OR genes with very low normalized counts is specific to OSNs, we analyzed single-cell RNA-seq data from 96 mouse T-helper lymphocytes^36^ and 288 single mouse embryonic stem cells^37^, captured using the same microfluidics method and in the same research facility as the 21 OSNs. We found examples of individual cells in both populations where up to 101 OR genes are expressed but, like the additional OR genes in the OSNs, they display very low normalized counts (Supplementary Fig. S5G).

In sum, 19 of 21 single mature OSNs express a single intact OR gene abundantly. They also express other OR genes but at three or more orders of magnitude less. We find no indication that these additional OR transcripts display coordinated patterns of expression, nor is low level OR gene expression specific to OSNs. These 19 mature OSNs thus obey the previously established one neuron - one receptor rule (reviewed in^32^,^33^,^38^).

### Monoallelic expression of OR genes

The OMP-GFP mouse strain was generated in the E14 embryonic stem cell line on a 129P2 genetic background, and then crossed to C57BL/6^15^. As OR genes have higher than average sequence variation between mouse strains^39^, we sought to identify the strain of origin for the OR genes that are abundantly expressed in single OSNs.

**Figure 6:**
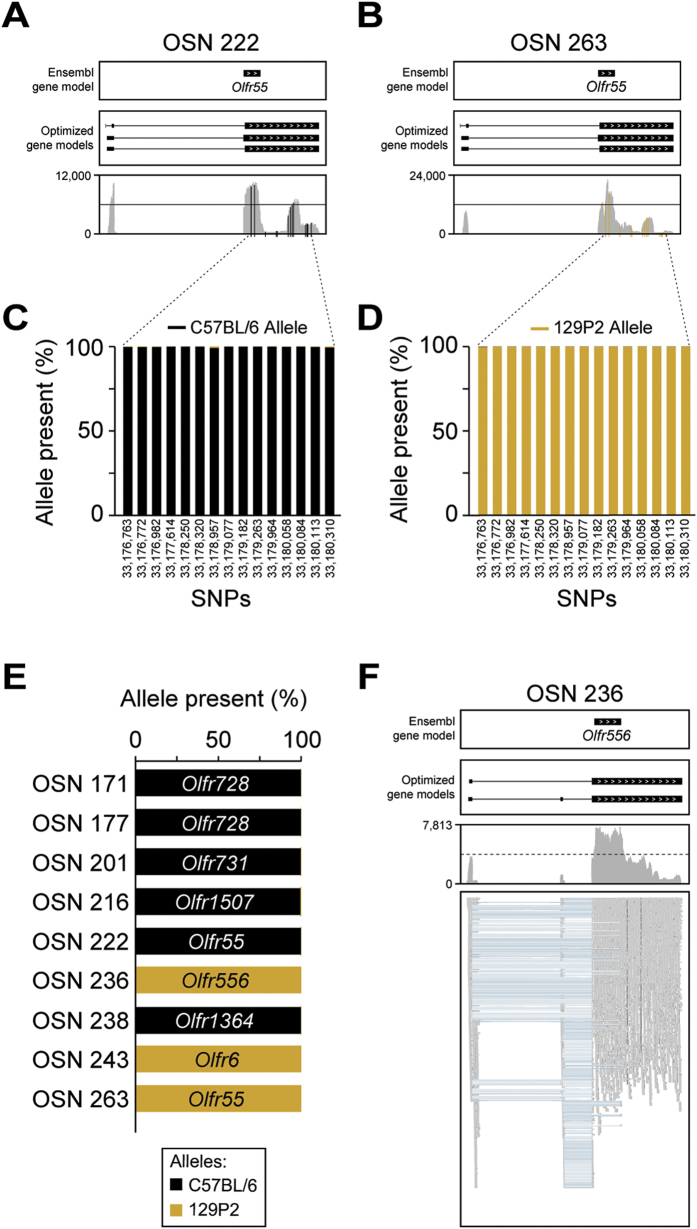
Monoallelic expression of OR genes in single OSNs. (**A,B**) Coverage plots of the sequencing data mapping to the same OR (*Olfr55*) in two cells, OSN 222 (**A**) and OSN 263 (**B**). Boxes correspond to exons and arrowheads indicate the strand of the gene. The existing Ensembl annotations are shown in the top box. Optimized OR gene models^11^ used in our analysis are shown in the middle box. The sequencing data mapping to the OR gene models is below. Reported single nucleotide polymorphisms (SNPs) used to map the C57BL/6 and 129P2 alleles are shown in black and golden vertical lines, respectively. (**C,D**) The percentage (%) of sequencing data showing the C57BL/6 (black) or the 129P2 (gold) allele at each SNP on *Olfr55* in OSN 222 (**C**) and OSN 263 (**D**). Genomic coordinates on chromosome 17 for each SNP are indicated in the x-axis. (**E**) The percentage (%) of sequencing data showing the C57BL/6 (black) or the 129P2 (gold) allele for all ORs with at least one reported informative SNP. In all cases, only one allele is expressed per cell. (**F**) Example of differential splicing of an OR gene. The sequencing reads in the bottom panel are represented by grey boxes; blue lines join portions of reads that map across exon junctions. Two isoforms are present, with the differential inclusion of the second exon. Thus individual OSNs express multiple isoforms of the chosen OR gene.

We first focused on *Olfr55,* an OR gene that is expressed abundantly in two cells, OSN 222 and OSN 263. The C57BL/6 and 129P2 strains differ by 15 SNPs within the *Olfr55* transcript (Fig. 6A,B). We quantified the number of reads from each allele at these positions in each *Olfr55*-expressing cell (Supplementary Fig. S6). Summed across the 15 SNPs, 54,563 of 54,677 reads (99.79%) in OSN 222 originate from the C57BL/6 allele of *Olfr55*, and 52,465 of 52,503 reads (99.93%) in OSN 263 are from the 129P2 allele (Fig. 6C,D). As both OSNs originated from the same individual mouse, these data demonstrate that monoallelic expression of *Olfr55* is extremely tightly regulated. Overall, of the 19 abundantly expressed intact OR genes, six expressed the C57BL/6 allele essentially exclusively (including both *Olfr728*-expressing OSNs) and three expressed the 129P2 allele (*Olfr6* and *Olfr556*, in addition to *Olfr55*). Both *Olfr6* and *Olfr556* are located near to the *Omp* locus on chromosome 7, suggesting their 129P2 allele has been maintained in the OMP-GFP strain for over 15 years of breeding by linkage disequilibrium (Fig. 6E). The 10 OR genes that are abundantly expressed in the remaining 10 single OSNs do not have SNPs that allow us to distinguish between the two strains.

We have shown previously that many OR genes have multiple transcripts within the WOM^11^, including some that, if translated, would alter the amino-acid sequence of the receptor and generate multiple protein isoforms. We do find multiple OR transcripts expressed within most single OSNs, such as the class I OR gene *Olfr556* in OSN 236 (Fig. 6F), but none that alter the coding sequence.

Taken together, we confirm and extend the rule of monoallelic expression^5^ of OR genes at the level of single OSNs, and show that it reaches a level of constraint that has thus far not been demonstrated.

### A neuronal type lacking OR gene expression

There is no abundantly expressed OR gene in two single cells, OSN 259 and OSN 261 (Figs 4,5C). These cells do express some OR genes, but again at very low levels: OR genes at best rank 3,752 (12.66 normalized counts) and 4,997 (7.7 normalized counts) respectively, which is similar to the OR gene expression levels in T-helper lymphocytes and ES cells (Supplementary Fig. S5G). Nor can we identify expression of any other known chemosensory receptor gene in these two cells (Supplementary Fig. S7A). They both also lack expression of *Adcy3* and *Cnga4*, components of the canonical OR-mediated signal transduction pathway, but express many other markers of mature OSNs such as *Omp*, *Gnal* and *Cnga2* (Fig. 4; and Supplementary Data S3). What are these cells?

**Figure 7:**
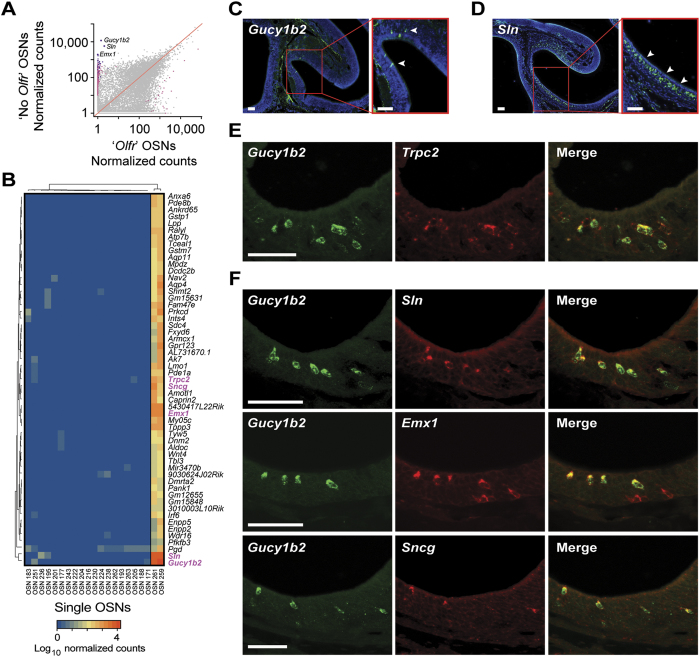
Gene expression profile of another neuronal type in the MOE. (**A**) Scatter plot of the mean expression values for all genes in the two cells lacking OR gene expression versus the remaining 19 OSNs. Statistically significant differentially expressed genes (FDR < 5%) are highlighted in pink; those that show consistent high expression in both cells lacking OR gene expression are in purple. The three most abundant DE genes are indicated (*Gucy1b2*, *Sln* and *Emx1*). (**B**) Heatmap of the 55 DE genes that constitute the gene expression signature of the cells lacking OR gene expression. (**C,D**) Cryosections of adult mouse MOE hybridized with cRNA probes for the two most highly expressed DE genes – *Gucy1b2* (**C**) and *Sln* (**D**). The hybridization signals are sparsely distributed within the MOE. (**E**) Two-color *in situ* hybridization of the top ranked marker (*Gucy1b2*) with *Trpc2*. As previously shown^18^, some, but not all of the *Trpc2* cells in the MOE also coexpress *Gucy1b2*. (**F**) Two-color *in situ* hybridization of the top ranked marker (*Gucy1b2*) with other differentially expressed genes, *Sln*, *Emx1* and *Sncg*. Arrowheads point to labeled cells. Scale bars, 50 μm.

What sets them apart from the other 19 OSNs, is that they express high levels of *Trpc2* (Fig. 4), a gene encoding a cation channel that was thought to be expressed exclusively in vomeronasal sensory neurons^40^. Recently *Trpc2* expression has been reported in two subsets of chemosensory neuron in the mouse MOE^17^. Comparing the transcriptomes of these two cells to the remaining 19, we find 494 genes that are classified as DE (Fig. 7A), including 55 that, similar to *Trpc2*, are only highly expressed in these two cells (Fig. 7B). To ascertain whether this number of shared DE genes is statistically meaningful, we carried out the same analysis for all 210 possible combinations of two cells among the 21 single OSNs. Intriguingly, this pair of cells (OSN 259 and OSN 261) share over twice the number of uniquely coexpressed genes than any other combination (Supplementary Fig. S7B), suggesting they do indeed represent two examples of a molecularly distinct type of neuron. The most abundant DE genes in these two cells are *Gucy1b2* (a soluble guanylyl cyclase), followed by *Sln* (sarcolipin, a transmembrane protein involved in mobilizing Ca^2+^ from the cytosol to the sarcoplasmic reticulum), and *Emx1* (a transcription factor involved in neuronal fate specification) (Fig. 7A,B). In comparison, *Trpc2* is only ranked 39^th^ by abundance among the DE genes. We used single-color *in situ* hybridization (ISH) to histologically characterize the MOE cells with this gene expression profile. We confirm that *Gucy1b2* is expressed in some cells within the mouse MOE, sparsely distributed within the OSN and sustentacular cell layers (Fig. 7C). *Sln* similarly identifies a subset of cells in the MOE (Fig. 7D). We then used two-color ISH to confirm that the *Gucy1b2*^*+*^ cells define a subset of the *Trpc2*^+^ cells in the MOE (Fig. 7E), consistent with our single-cell RNA-seq data (Fig. 7B) and recent reports^17^,^18^. We used a similar approach to determine whether the other most differentially expressed genes identified by RNA-seq identify the same subset of cells. We find that *Sln* and *Gucy1b2* are coexpressed and that many, but not all, of the cells that express *Emx1* also express *Gucy1b2* (Fig. 7F), suggesting further molecular subdivisions may exist within the OMP^+^ neurons of the MOE. Finally we selected a less-abundantly expressed gene, *Sncg* (γ-synuclein), ranked 7^th^ by abundance among the 55 DE genes, and find that this gene is also coexpressed with *Gucy1b2* (Fig. 7F).

Thus, these two cells appear to be examples of the recently discovered type B Trpc2^+^ cells in the MOE, which we here firmly establish as a neuronal cell type that is fundamentally distinct from canonical OSNs.

## Discussion

We have here applied RNA-seq hierarchically to the main olfactory system of the mouse, starting with WOM (crude tissue samples that can be dissected from the nasal cavity and contain non-olfactory cell types), proceeding to GFP^+^ cell pools that were FACS-sorted from heterozygous OMP-GFP mice (mature OSNs), further to sorted OMP-GFP^low^ and OMP-GFP^high^ subpopulations, finally arriving at single OMP-GFP^high^ OSNs. These samples all originated from heterozygous OMP-GFP gene-targeted mice in a mixed 129P2 × C57BL/6 background^15^. Our data afford six conclusions.

First, we show that 1,087 from 1,099 (98.9%) intact OR genes are expressed in mature OSNs, indicating that essentially every member of the OR gene repertoire is a candidate receptor for odorants based on the minimal criterion of expression in mature OSNs. Second, the OR gene expression levels in sorted OSNs are highly correlated to their levels in WOM. This proportionality proves that our methods of tissue dissociation and FACS retrieve and isolate these different OR-expressing OSN subsets from the MOE in a reliable and representative manner - a critical experimental requirement that thus far had not been demonstrated. Third, we uncover a hitherto unknown bipartition between OMP-GFP^low^ and OMP-GFP^high^ OSNs. We show that these subsets represent a subdivision within the population of mature OSNs, with OMP-GFP^low^ cells being somewhat less mature than OMP-GFP^high^ cells. Fourth, we find that 19 of 21 single OMP-GFP^high^ OSNs each express a single intact OR gene abundantly, providing the most direct evidence for the one neuron - one receptor rule to date. Interestingly, these 19 cells each coexpress a variety of other OR genes at low levels, but so do non-olfactory cells like T-helper lymphocytes and embryonic stem cells, thereby providing a warning for unvalidated interpretation of low-level coexpression of OR genes in OSNs. Fifth, we demonstrate that monoallelic expression of the OR gene that is abundantly expressed in an OSN is extremely tight in the 9 of 19 OSNs where SNPs enable discrimination between the two alleles in the mixed 129P2 × C57BL/6 background of the OMP-GFP strain. Sixth, the remaining two single OMP-GFP^high^ OSNs appear to be examples of the recently discovered type B Trpc2^+^ MOE cells^17^,^18^,^19^. Our identification of 53 upregulated genes in addition to *Trpc2* and *Gucy1b2* firmly establishes type B Trpc2^+^ MOE cells as a novel type of chemosensory neuron within the MOE that is fundamentally distinct from canonical OSNs. Our hierarchical strategy has thus proven that it can identify and classify a minor MOE cell type, paving the way for identification of additional MOE cell types in an unbiased, explorative, discovery-based approach.

Advances in RNA sequencing technologies have made possible the fast and cost-effective whole transcriptome profiling of organs, tissues, and now single cells. We and others have recently applied RNA-seq to the mouse olfactory system, allowing us to generate complete lists of the average expression level for each annotated gene across all cell types that are contained within the crude tissue samples that can be dissected from the nasal cavity^11^,^41^,^42^. But this approach does not allow discrimination between sets of genes variably expressed in different cell types within a tissue, and does not enable exploration of molecular heterogeneity within a particular cell type, such as mature, canonical OSNs. The nervous system is especially sensitive to these issues, due to its vast functional diversity of neurons and supporting cells. A growing body of work has addressed cellular diversity by sequencing RNA extracted from pools of neurons that express a shared, single marker^43^. More recently, single neurons from specific brain areas have been sampled for RNA-seq, enabling subclassification of neuronal types by gene expression profiles^44^,^45^,^46^.

OSNs constitute an extreme challange for the molecular subclassification of neurons by RNA-seq. Since all mature OSNs are likely to fulfill the same general role in chemoreception and are distinguished only by the molecular identity of the odorants that they detect, it is widely accepted that expression of a single allele of a single intact OR gene is a functional molecular classifier. Different subsets of OSN can thus be identified, molecularly compared, and contrasted in the context of the expressed OR gene. Two early studies analyzed RNA from nine single OSNs using microarrays and LongSAGE, but no data on OR gene expression were reported most likely due to extensive 3’ bias in cDNA amplification^31^,^47^. Here we have embarked on a systematic program to deconstruct the molecular heterogeneity of mouse OSNs. By combining RNA-seq with FACS in a tiered fashion, we have sampled the entire transcriptome of over 99.4% of the population of canonical OSNs. Focusing on the most mature subpopulation (OMP-GFP^high^), single-cell RNA-seq analysis allowed us to directly test the widely held paradigms of monogenic and monoallelic OR gene expression, and enabled us to characterize a minor type of MOE neurons that do not express OR genes or other known chemoreceptor genes.

Most OR genes remain candidate receptors for odorants because no odorous ligands have been identified. Arguably a minimal criterion for a gene that phylogenetically belongs to the OR gene repertoire to serve as a receptor for odorants, is that it must be expressed in an olfactory epithelium such as the MOE. This issue has become more relevant in light of increasing evidence of ectopic (non-olfactory) expression of OR genes^48^. We here report evidence for expression of 98.9% of intact OR genes in mature OSNs. We obtained no reads in our OSN samples for only six intact OR genes (*Olfr247*, *Olfr331*, *Olfr456*, *Olfr663*, *Olfr1008*, *Olfr1128*) that are nonetheless expressed in our WOM samples, albeit at extremely low levels. This minor discrepancy suggests that OSNs that express these OR genes are sparse and/or do not mature to the OMP^+^ stage, possibly because the genes do not encode a functional OR. On the other hand, this discrepancy between WOM and OSNs also emphasizes the importance of very deep sampling of cells for the study of the complete OSN population. Using conservative statistical thresholds, our analysis identified 29% more genes enriched in OSNs and 300% more genes enriched in other WOM cell types compared to those reported by Sammeta *et al.*^21^. Some genes that are enriched in OSNs with high abundance have been previously identified and characterized through other enrichment methods^49^,^50^, but others have not yet been reported as being expressed in OSNs.

OMP is widely used as a marker of most mature OSN types^23^. Instead of observing cells with a continuum of GFP expression levels by FACS of heterozygous OMP-GFP mice, we were intrigued to find two distinct subpopulations of GFP^+^ cells. Both subpopulations represent mature OMP^+^ OSNs by all previously known molecular markers. It is possible that these subpopulations are distinguished by post-transcriptional differences in proteins involved in OR gene choice and stabilization^25^,^26^ or axon guidance^27^, but we observed only very minor differences in the expression of these genes. Instead, the set of genes that do distinguish them suggests that their constituent OSNs are at different stages during late neuronal maturation, with OMP-GFP^low^ cells being somewhat less mature than OMP-GFP^high^ cells. It appears that OSNs increase *Omp* RNA levels during late maturation. We speculate that a discrete and reasonably rapid event must be occurring during the transition between the OMP-GFP^low^ and OMP-GFP^high^ subpopulations in adult mice, such as completion of successful and stable innervation of a glomerulus that is appropriate for the expressed OR. A recent analysis of OSN maturation in postnatal day 7 mice^51^ revealed the temporal sequence of expression *Gap43*, OR, *Adcy3*, and *Omp*, but did not indicate a distinction between OMP-GFP^low^ and OMP-GFP^high^. Conceivably this distinction cannot be made histologically. Picking single OMP^+^ OSNs with a pipette prior to single-cell RNA-seq may also suffer from the inability to distinguish between the OMP-GFP^low^ and OMP-GFP^high^ subpopulations. Until proven otherwise, we consider it prudent to select OSNs from the most mature, OMP-GFP^high^ population for single-cell RNA-seq analysis, in order to minimize the likelihood of sampling less-mature OSNs, which may include OSNs that coexpress two or more OR genes abundantly.

It is now widely accepted, though has been difficult to prove directly, that each mature OSN expresses a single intact OR gene from a single allele^4^,^5^,^32^,^33^,^35^,^38^. A hierarchy of events, some stochastic others deterministic, appear necessary to generate such exquisite specificity and restriction of gene expression^38^,^52^, but how these processes are coordinated at the molecular level remains largely unknown. Recent advances in single-cell transcriptomics^53^ now permit us to quantify the transcriptomes of single OSNs, including all OR genes, and to directly assess their molecular heterogeneity at a level that was not previously possible.

We first searched for genes with a high coefficient of variation (CV) across cells and, as expected, identified OR genes but also other orphan GPCR genes and a statistical enrichment in zinc-finger protein genes. In each of the OSNs that passed our stringent quality control process, we found between 11 and 28 OR genes with evidence of transcription per cell; but in most cases we estimate that a single abundant OR accounts, on average, for 98.1% of the sequencing data mapped to OR genes. Compared to the classical evidence for the one neuron - one receptor rule, which was generated by single-cell RT-PCR using degenerate primers for OR genes^4^ and other lines of evidence^33^, we argue that our single-cell RNA-seq data provide the clearest direct evidence in support of this paradigm.

What consequence might the low-level coexpression of other OR genes have? Single-cell RNA-seq analysis of two other non-olfactory cell types, T-helper lymphocytes and embryonic stem cells, reveals similar patterns of low-level OR gene transcription, suggesting it may be a feature of many cell types and not functionally relevant for OSNs. Moreover, bulk RNA-seq analysis has revealed that genes expressed at very low levels are not associated with active chromatin markers and lack correlative protein expression data^54^.

Nevertheless, we cannot discount the possibility that one or more of the additional OR genes could generate low levels of functional OR protein. Moreover we sampled less than 1.6% of the subsets of OSN, and our strict quality control procedure selected against the inclusion of OSNs potentially expressing more than one OR at high levels, because these cannot be distinguished empirically from cross-contamination, even when only one cell is visible in the Fluidigm microfluidic chip^46^. New technologies for single-cell transcriptome analysis continue to emerge, enabling tens of thousands of neurons to be analyzed in parallel^46^,^55^. A comprehensive sampling on this scale will be necessary to fully assess the pervasiveness of the one neuron – one receptor rule in OSNs.

By capturing cells from a single mouse on a mixed genetic background, we could use SNP analysis to quantify allele-specific OR gene expression for approximately half of the single OSNs. In all cases we find that monoallelic expression is extremely tightly regulated. Together, these data provide a novel insight into the extraordinary stringency of transcriptional control of OR expression at single-cell resolution. It is likely that allelic choice or silencing in OR genes is the first restrictive event to occur, perhaps even in progenitor cells prior to the development of the olfactory system^52^. We find that this process is extremely strongly maintained in mature OSNs (>99.7%). In contrast, the promoter and/or splicing machinery of an active OR allele appears to be under weaker regulatory control, permitting in some cases multiple OR gene transcripts from the same allele often in roughly equal abundances. All of the transcript variation we detected is within the untranslated regions (UTRs), most likely resulting in no functional consequence for the OR protein and hence exerting no pressure to ensure a single transcript is produced. However variation in UTRs can influence the amount of OR protein produced by altering the stability, localization, or translational efficiency of the transcript^56^.

In addition to the canonical OSNs, which express OR genes, small subsets of neurons that express other chemoreceptor types have been reported in the mouse MOE^12^,^13^,^57^,^58^. The subset of mature OSNs that express *Gucy2d* expresses little or no OMP^16^. Our experimental design precludes the discovery of further chemosensory cell types that do not express OMP at all, or at low levels. However other minor cell types express OMP and therefore would be expected to be represented in the OSN pools or in the mature, OMP-GFP^high^ subpopulation. Full transcriptome analysis of single OSNs revealed two that represent a molecularly distinct type, but are not TAAR-expressing neurons. Instead, these appear to be examples of the recently described type B Trpc2^+^ MOE cells^17^,^18^,^19^. Consistent with these recent reports, these two OSNs lack expression of *Adcy3* but abundantly express *Gucy1b2* and *Trpc2*. Importantly, we identified an additional 53 genes not expressed in the canonical OSNs but expressed in both type B cells, and validated three as markers of these cells by ISH. It remains puzzling that two out of 21 single captured OSNs (~10%) represent a minor population of OMP^+^ cells in the MOE: a 3-week old mouse has only 16,115 of these type B Trpc2^+^ MOE cells^18^, which is a mere 0.24% of the estimated ~6.6 million OR-expressing OSNs at the same age^59^. We speculate that the superficial location of type B Trpc2^+^ MOE cells within the sustentacular layer may facilitate dissociation of these cells. Alternatively, these cells may have a different shape or size compared to canonical OSNs, more amenable to capture by the microfluidic device. Are these neurons chemosensory? Interestingly, they do not express an OR gene abundantly, nor any other previously described chemoreceptor. However, they express many of the genes involved in olfactory signal transduction and project axons to form glomeruli^17^,^18^. Further work will be necessary to determine whether, analagous to the role of *Gucy2d* in another minor type of OSN^60^, *Gucy1b2* itself serves a chemosensory function in these cells.

## Methods

### Mice

Mice heterozygous for the OMP-GFP (B6:129P2-Omp^tm3Mom/MomJ^, The Jackson Laboratory, Stock # 006667) mutation^15^ were used in RNA-seq experiments, and C57BL6/J mice were used for *in situ* hybridization experiments. For the experiments done in the UK, mice were maintained in accordance with UK Home Office regulations, under a project license approved by the Wellcome Trust Sanger Institute Animal Welfare and Ethical Review Body. Experiments done in Germany were carried out in accordance with the German Animal Welfare Act, European Communities Council Directive 2010/63/EU, and the ethical and animal welfare guidelines of the Max Planck Institute of Biophysics and the Max Planck Research Unit for Neurogenetics, with approval from the Regierungspräsidium Darmstadt and the Veterinäramt of Frankfurt.

### Capture, library preparation and sequencing of whole olfactory mucosa and pools of GFP^+^ OSNs

Whole olfactory mucosa (WOM) was dissected from three 21-day-old OMP-GFP heterozygous mice (two males and one female), and homogenized in Lysis RLT Buffer. Total RNA was extracted using the RNeasy Mini kit (QIAGEN) together with genomic DNA eliminator (QIAGEN) according to manufacturer’s protocol. To obtain pools of 10 million OMP-GFP^+^ OSNs, a total of 45 male and female, 25-day-old OMP-GFP heterozygous mice were used in three sorting experiments on three separate days: 14 (OSN1), 16 (OSN2), and 15 (OSN3) mice, respectively. WOM was dissected from olfactory turbinates and septum, and includes the septal organ but not the VNO. Tissue was collected in HBSS without Ca^2+^ and Mg^2+^ (Gibco) on ice. Collected tissue was minced with scissors followed by enzymatic digestion in HBSS without Ca^2+^ and Mg^2+^ supplemented with 44 U/ml Dispase (Invitrogen), 1000 U/ml Collagenase type II (Invitrogen) and 10 mg/ml DNaseI (Roche), for 20–30 min at 37 °C with agitation. Digested tissue was centrifuged at 0.4–0.5 × 1000 rcf for 5 min and washed in HBSS without Ca^2+^ and Mg^2+^. Dissociated cell suspension was passed through a 70 μm cell strainer (Falcon) into a sterile 35 × 10 mm petri dish (Falcon). Propidium iodide (final concentration of 1 μg/ml) was added to the final cell suspension prior to sorting. OMP-GFP^+^ cells were sorted by flow-cytometry using a JSAN desktop sorter (Bay bioscience Kobe, Japan) equipped with a 488 nm solid-state laser (DPSS) at 20 mW. A total of 10 million OMP-GFP^+^ cells were collected directly in Lysis RLT buffer. The time elapsed between mouse euthanasia and the collection of the cells into lysis buffer was ~1 hr. Total RNA was extracted using RNeasy Micro kit (QIAGEN) together with genomic DNA eliminator (QIAGEN) according to manufacturer’s protocol. mRNA was prepared for sequencing using the TruSeq RNA sample preparation kit (Illumina) with a selected fragment size of 200–300 bp. All six samples (three WOM and three OSN samples) were multiplexed together and sequenced across two lanes on an Illumina HiSeq 2000, to generate paired-end 100 bp sequencing reads.

### Capture, library preparation, and sequencing of OMP-GFP^low^ and OMP-GFP^high^ OSNs

Male OMP-GFP heterozygous mice aged 15, 24 and 25 weeks were used in three sorting experiments on three separate days. WOM was dissected from olfactory turbinates and septum. Tissue was collected in PBS without Ca^2+^ and Mg^2+^ (Gibco) on ice. Collected tissue was finely minced with forceps in 1 mL ice-cold dissociation buffer (1.1 mM EDTA; 5.5 mM DL-Cysteine-HCl; Papain 2.2 U/mL in PBS without Ca^2+^ and Mg^2+^), and incubated for 15–20 min at 37 °C with agitation. An equal volume of DNAse solution (50 U/mL of DNAse I and DNAse buffer in PBS, Roche) was added to the dissociated cell suspension, gently triturated, and diluted 5-fold with pre-heated (37 °C) phenol-red-free DMEM (Gibco) plus 4% fetal bovine serum (Sigma) (hereafter referred to as ‘media’). After centrifugation at 1000 rpm for 5 min, the supernatant was removed, the cell pellet re-suspended in 500 μl of media and passed through a 30 μM mesh filter (Partec). The cell suspension was then kept on ice, and GFP-positive cells were immediately isolated using a MoFlo™ XDP cell sorter (Beckman Coulter, Inc).

A total of 10,000 OMP-GFP^low^ and 10,000 OMP-GFP^high^ cells sorted from the same mouse were collected in a 1.5 ml Eppendorf tube, immediately frozen in dry-ice and kept at −80 °C. The time elapsed between mouse euthanasia and the termination of the FACS procedure was ~1 hour. RNA was extracted using the RNeasy Plus Micro Kit (Qiagen), together with genomic DNA eliminator (QIAGEN), according to the manufacturer’s instructions. Reverse transcription and cDNA pre-amplification were performed using the SMARTer PCR cDNA Synthesis kit (Clontech) and the Advantage 2 PCR kit (Clontech) following the instructions in Appendix 1 of the Fluidigm manual. cDNA was harvested and quantified with the Bioanalyzer DNA High-Sensitivity kit (Agilent Technologies). Nextera libraries were prepared using the Nextera XT DNA Sample Preparation Kit and the Nextera Index Kit (Illumina) following the instructions in the Fluidigm manual. Multiplexed libraries were pooled, and paired-end 100-bp sequencing was performed on one flow–cell (two lanes) of an Illumina HiSeq 2500.

### Capture, library preparation and sequencing of single OSNs

One male OMP-GFP heterozygous mouse aged 23 weeks was used in one sorting experiment. WOM dissection and dissociation procedures were the same as in the previous section. 5,000 OMP-GFP^high^ cells were isolated by FACS and loaded onto a 10–17 μm C1 Single-Cell Auto Prep IFC chip (Fluidigm). Cell capture was performed according to the manufacturer’s instructions. The capture efficiency was verified by visual inspection using a fluorescent microscope; there were single fluorescent cells with neuron-like morphology in 58 wells, more than one cell (or one cell plus debris) in 30 wells, and no cells in 8 wells. Upon capture, reverse transcription and cDNA pre-amplification were performed in the 10–17 μm C1 Single-Cell Auto Prep IFC using the SMARTer PCR cDNA Synthesis kit (Clontech) and the Advantage 2 PCR kit (Clontech). One μl of the ERCC Spike-In Control Mix (Ambion) in a 1:10,000 dilution in C1 Loading Reagent was added to the lysis mix for the OSNs. cDNA was harvested and quantified with the Bioanalyzer DNA High-Sensitivity kit (Agilent Technologies). Nextera libraries were prepared using the Nextera XT DNA Sample Preparation Kit and the Nextera Index Kit (Illumina) following the instructions in the Fluidigm manual. Multiplexed libraries were pooled, and paired-end 100-bp sequencing was performed on one flow–cell (two lanes) of an Illumina HiSeq 2500.

### RNA-seq data processing and alignment

Sequencing data were aligned with STAR 2.3^61^ to the GRCm38 mouse reference genome plus the ERCC spike-in sequences, with options*–outFilterMultimapNmax 1000–outFilterMismatchNmax 4–outFilterMatchNmin 100–alignIntronMax 50000–alignMatesGapMax 50500–outSAMstrandField intronMotif–outFilterType BySJout*. The genome annotation used was from the Ensembl mouse genome database, version 72 (http://jun2013.archive.ensembl.org/info/data/ftp/index.html). The GTF file was modified to replace all the gene models for olfactory and vomeronasal receptors with those reported in^11^. Additionally, the set of transcripts reported for *Trpc2* contain both short and long isoforms of the gene; the long isoforms represent a fusion with a different gene and were therefore removed (ENSMUST00000084843, ENSMUST00000094129, ENSMUST00000094130, ENSMUST00000106950, ENSMUST00000123372, ENSMUST00000125197, ENSMUST00000139104, ENSMUST00000140395, ENSMUST00000141646, ENSMUST00000142629, ENSMUST00000143839, ENSMUST00000146450, ENSMUST00000153176). Finally, the gene *Gm20715*, a predicted gene that undergoes nonsense mediated decay, was also removed from the GTF file because it overlaps with *Olfr1344*; this overlap causes all the reads aligned to the OR to be deemed ambiguous. BAM files were processed using SAMtools^62^.

### Gene expression estimation and data analysis

The number of fragments uniquely aligned to each gene was obtained using the HTSeq 0.6.1 package, with the script *htseq-count*, mode *intersection-nonempty*^63^. All multi-mapped fragments were discarded. Raw counts were normalized to account for sequencing depth between samples, using the procedure implemented in the DESeq2 package^64^. For the single-cell data, ERCC spike-ins were not included for data normalization. Normalized counts for all datasets analyzed are provided in Supplementary Data S1-3. Data analysis, statistical testing and plotting was carried out in R (http://www.R-project.org); all the heatmaps were produced with the gplots package using the log_10_ transformed normalized counts + 1^65^. To deconvolve bimodal distributions into two normal-like distributions (Fig. 5B, Supplementary Fig. S3A), Gaussian mixture models were used, through the expectation-maximization algorithm of the mixtools Bioconductor package^66^.

### Differential expression analysis

To test for differential expression we used DESeq2 1.4.5 with standard parameters. When applied to the single-cell data, the parameter *minReplicatesForReplace* was set to *Inf* to turn off the automatic outlier replacement. Genes were considered differentially expressed if they had an adjusted p-value of 0.05 or less (equivalent to a false discovery rate of 5%). All results from the DE analyses are provided in Supplementary Data S1-3; the columns contain the following data: *baseMean* corresponds to the mean normalized expression value for the gene across all samples; *log2FoldChange* is the fold change between the two groups tested, log_2_ transformed; *lfcSE* corresponds to the standard error associated with the fold change estimation; *stat* is the Wald statistic; *pvalue* is the p-value of the test; and *padj* is the p-value after adjusting for multiple testing (Benjamini-Hochberg). Genes that have both their *pvalue* and *padj* set to NA contain outliers; genes with only their *padj* set to NA were filtered prior to the test because their normalized counts were too low.

### Allelic discrimination of OR genes

To determine the allele expressed for each OR, the Mouse Genomes Project database release 1410 was queried (http://www.sanger.ac.uk/sanger/Mouse_SnpViewer/rel-1410)^67^ to obtain all the SNPs for 129P2 that overlap OR gene models. These positions were visualized on IGV and the numbers of fragments containing each nucleotide were extracted.

More information is available in the Supplementary Methods.

## Additional Information

**How to cite this article**: Saraiva, L. R. *et al.* Hierarchical deconstruction of mouse olfactory sensory neurons: from whole mucosa to single-cell RNA-seq. *Sci. Rep.* **5**, 18178; doi: 10.1038/srep18178 (2015).

## Supplementary Material

Supplementary Information

Supplementary Dataset 1

Supplementary Dataset 2

Supplementary Dataset 3
