# Supplementary material for: Hierarchical deconstruction of mouse olfactory sensory neurons: from whole mucosa to single-cell RNA-seq
Source: Sci Rep. 2015 Dec 16;5:18178. doi: 10.1038/srep18178 (PMC4680959; doi:10.1038/srep18178)
Supplement: Supplementary Information [file srep18178-s1.pdf]

Hierarchical deconstruction of mouse olfactory sensory neurons: from whole mucosa to single-cell RNA-seq

Luis R. Saraiva[1,2,3,+], Ximena Ibarra-Soria[1,+], Mona Khan[4], Masayo Omura[4], Antonio Scialdone[1,2], Peter Mombaerts[4], John C. Marioni[1,2,#,*], and Darren W. Logan[1,5,#,*]

1, Wellcome Trust Sanger Institute, Wellcome Genome Campus, Hinxton-Cambridge, CB10 1SD, United Kingdom

2, European Bioinformatics Institute (EMBL-EBI), European Molecular Biology Laboratory, Wellcome Genome Campus, Hinxton-Cambridge, CB10 1SD, United Kingdom

3, Department of Experimental Genetics, Sidra Medical & Research Center, Qatar Foundation, PO Box 26999, Doha, Qatar

4, Max Planck Research Unit for Neurogenetics, Max-von-Laue-Strasse 4, 60438 Frankfurt, Germany

5, Monell Chemical Senses Center, 3500 Market Street, Philadelphia, PA 19104, USA

+, These authors contributed equally to this work

#, These authors jointly supervised this work

*, Correspondence and requests for materials should be addressed to D.W.L. (email: dl5@sanger.ac.uk) or J.C.M. (email: marioni@ebi.ac.uk)

Contact

Corresponding author: Darren W. Logan

Wellcome Trust Sanger Institute, Wellcome Genome Campus,

Hinxton-Cambridge, CB10 1SA, United Kingdom

Tel: +44 1223 496854, E-mail: dl5@sanger.ac.uk

Supplementary figures

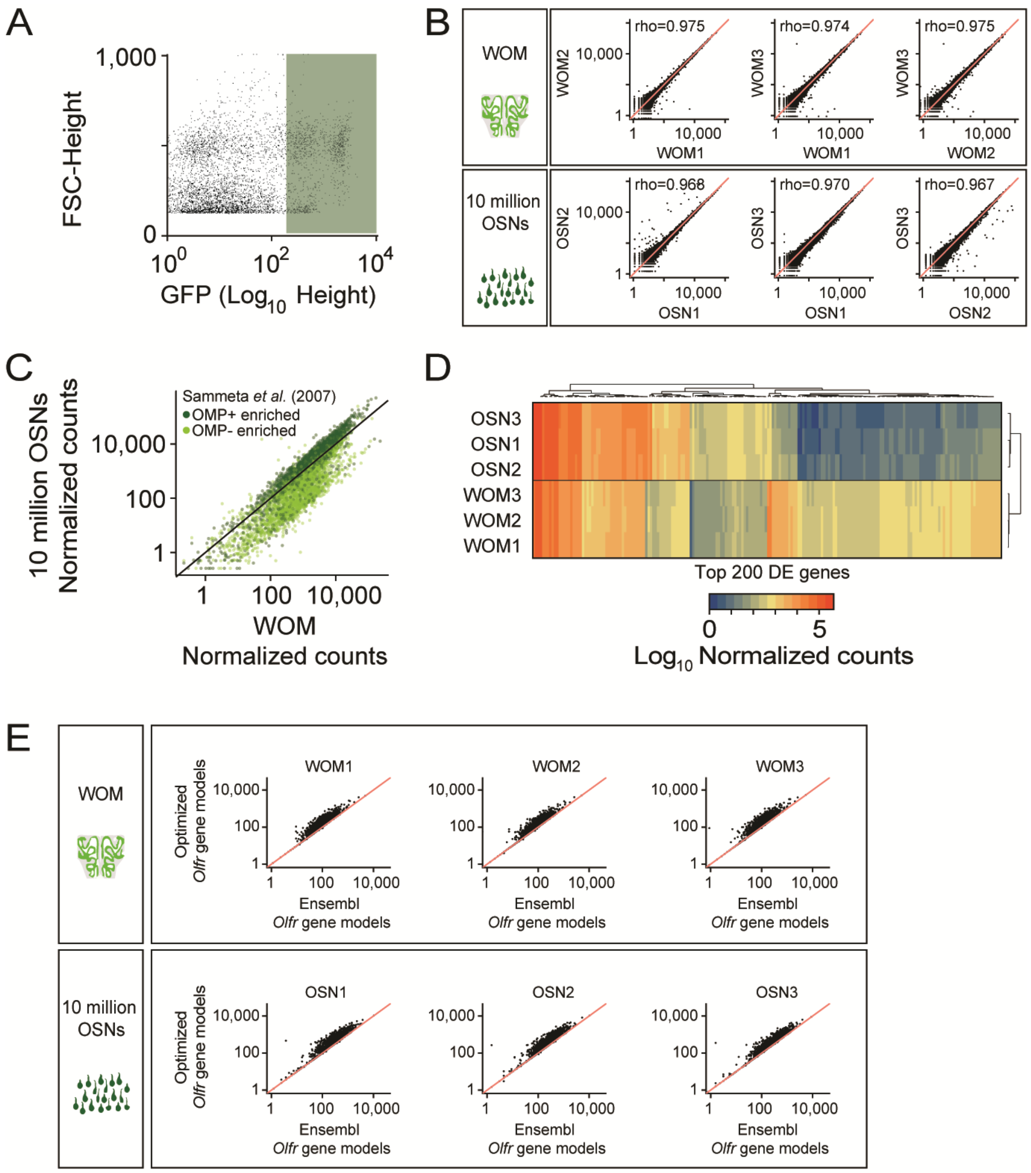

Supplementary Figure S1, related to Figure 1 – The transcriptomes of whole olfactory mucosa (WOM) and pools of 10 million OSNs. (A) WOM samples of OMP-GFP (+/-) mice were dissociated, and pools of 10 million GFP+ cells (OSNs) were collected by FACS gating based on fluorescence intensity (dark green shading). (B) Scatter plots of the expression levels of all genes in three biological replicates of WOM and OSNs. Spearman correlation coefficients were calculated and the rho values are indicated. The red line indicates the 1:1 diagonal. (C) Scatter plot of the expression levels for genes that were previously reported to be enriched in the OMP+ (dark green dots) or OMP- (light green dots) fractions of WOM samples from the same strain of OMP-GFP mice [19], here in WOM versus OSNs. Genes enriched in the OMP- fraction are more abundant in WOM, while genes enriched in the OMP+ fraction are more abundant in OSNs. The black line indicates the 1:1 diagonal. (D) Heatmap displaying the top 200 differentially expressed (DE) genes between WOM and OSNs. (E) Scatter plots of the expression levels of the OR genes as estimated by using the gene models annotated in Ensembl (x-axis) or in Ibarra-Soria *et al.* (2014) (y-axis), for each WOM and OSN sample. The red line indicates the 1:1 diagonal. Gene expression estimates are considerably higher when the optimized gene models from Ibarra-Soria *et al.* (2014) are used.

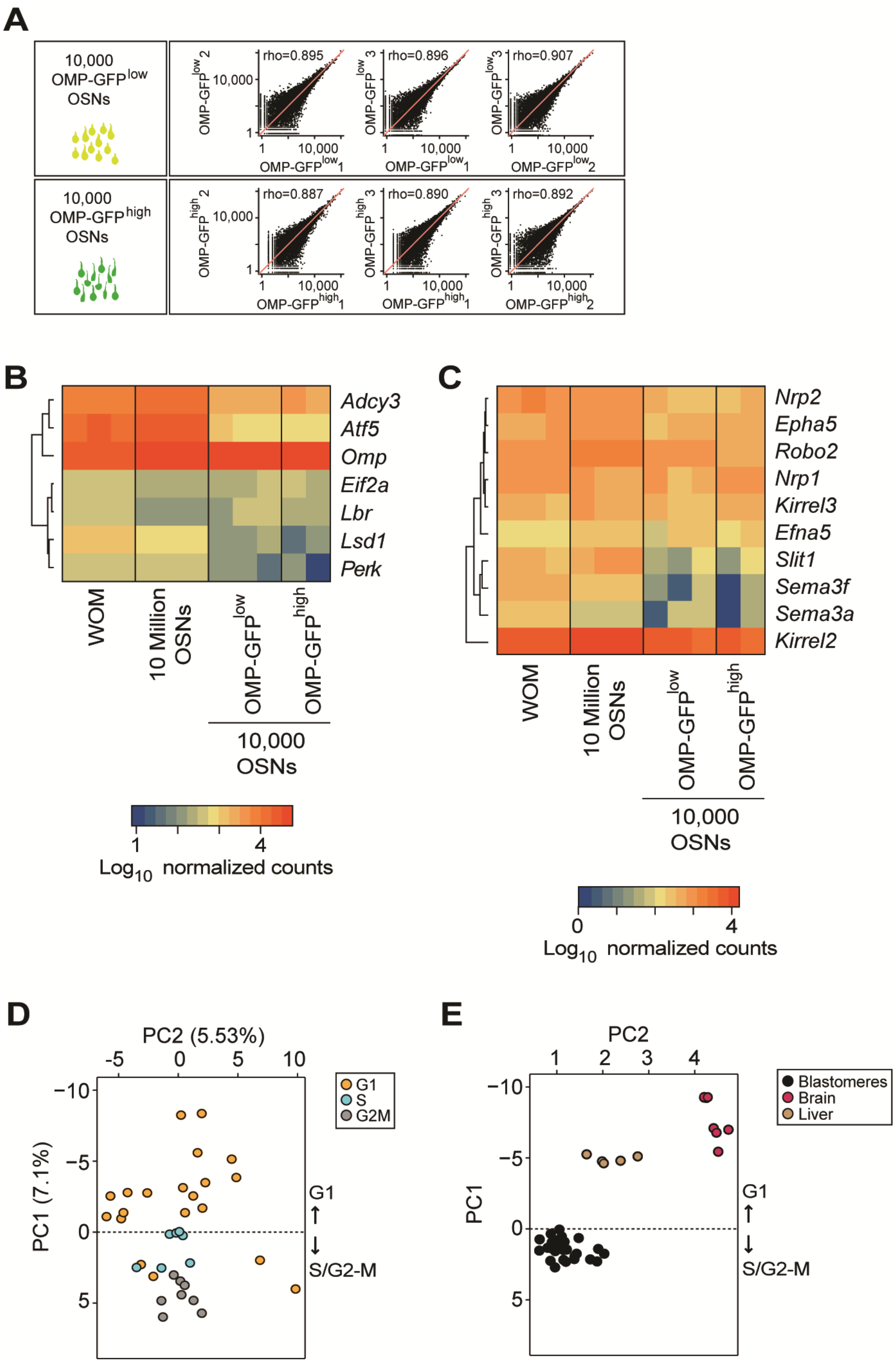
A
10,000 OMP-GFP low OSNs
10,000 OMP-GFP high OSNs
rho=0.895
rho=0.896
rho=0.907
rho=0.887
rho=0.890
rho=0.892
OMP-GFP low 1
OMP-GFP low 2
OMP-GFP low 3
OMP-GFP high 1
OMP-GFP high 2
OMP-GFP high 3
B
Adcy3
Atf5
Omp
Eif2a
Lbr
Lsd1
Perk
WOM
10 Million OSNs
OMP-GFP low
OMP-GFP high
10,000 OSNs
Log10 normalized counts
C
Nrp2
Epha5
Robo2
Nrp1
Kirrel3
Efna5
Slit1
Sema3f
Sema3a
Kirrel2
WOM
10 Million OSNs
OMP-GFP low
OMP-GFP high
10,000 OSNs
Log10 normalized counts
D
PC2 (5.53%)
PC1 (7.1%)
G1
S
G2M
G1
S/G2-M
E
PC2
PC1
Blastomeres
Brain
Liver
G1
S/G2-M

Supplementary Figure S2, related to Figure 2 – The transcriptomes of OMP-GFP^{low} and OMP-GFP^{high} OSNs. (A) Scatter plots of the expression levels of all genes in the biological replicates of OMP-GFP^{low} and OMP-GFP^{high} OSNs. Spearman correlation coefficients were calculated and the rho values are indicated. (B) Heatmap of the expression levels of genes involved in establishing monogenic expression in OSNs [20,21]. No significant differences are observed between the OMP-GFP^{low} and OMP-GFP^{high} populations. (C) Heatmap of the expression levels of some genes involved in OSN axon guidance [22]. Only *Robo2* is significantly different between the OMP-GFP^{low} and OMP-GFP^{high} populations. (D) Principal Component Analysis (PCA) on ES cells (training dataset) shows that the first principal component (PC1) approximately tracks the cell-cycle phase. In particular, G1 cells (except a few outliers) have a negative value on PC1, while the PC1 values of S and G2-M cells are positive. (E) Projection of the blastomeres (single-cells), brain (bulk) and liver (single-cells) samples onto the cell cycle PCA that differentiates between cell cycle stages (see Methods for details). PC1 segregates the samples based on their cell cycle stage, with samples in G1 having negative values and samples in S/G2-M positive values.

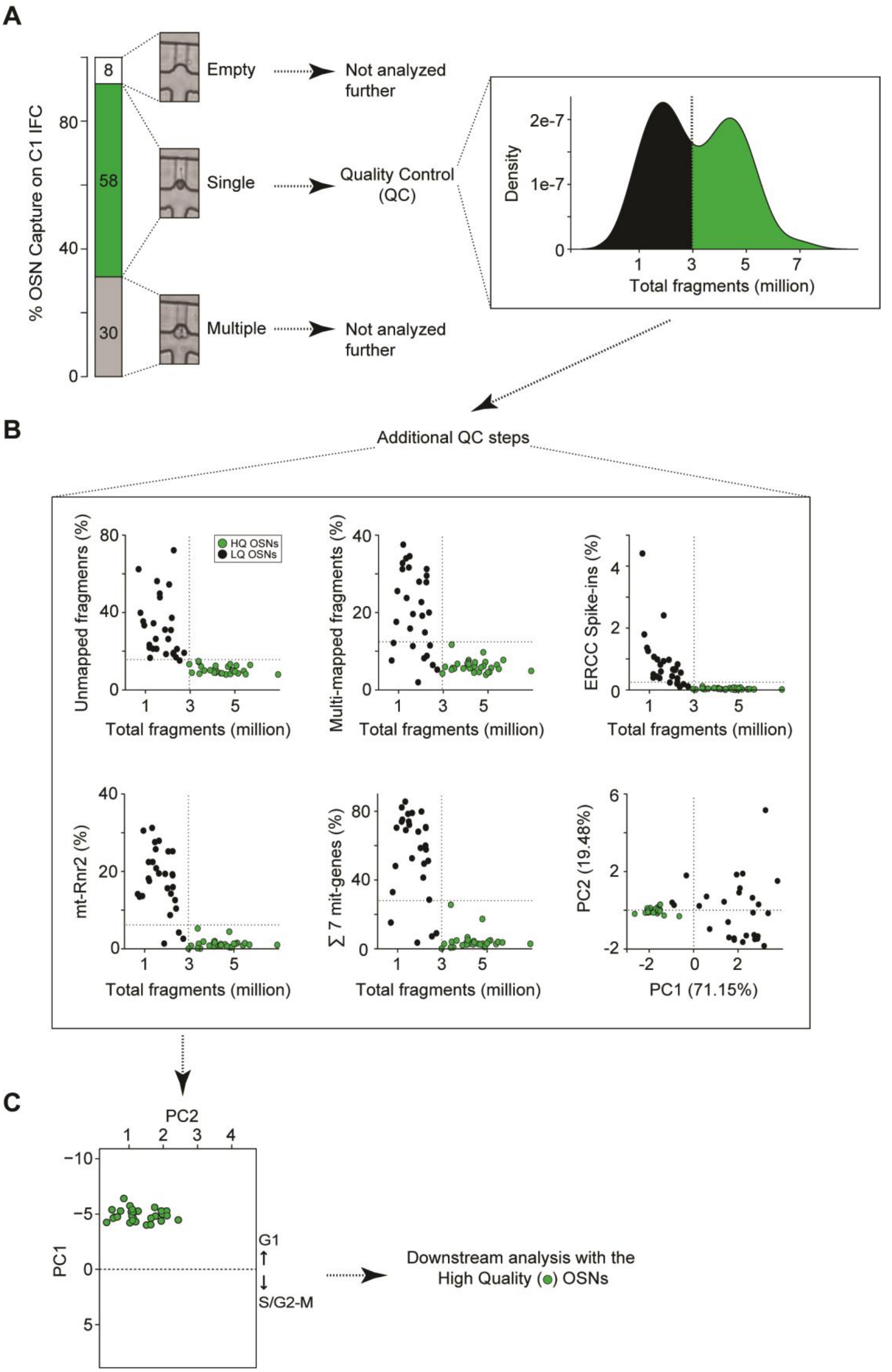
A
% OSN Capture on C1 IFC
8
58
30
Empty
Single
Multiple
Not analyzed further
Quality Control (QC)
Not analyzed further
Density
2e-7
1e-7
Total fragments (million)
B
Additional QC steps
HQ OSNs
LQ OSNs
Unmapped fragmenrs (%)
Multi-mapped fragments (%)
ERCC Spike-ins (%)
mt-Rnr2 (%)
Σ 7 mit-genes (%)
Total fragments (million)
PC2 (19.48%)
PC1 (71.15%)
C
PC2
PC1
G1
S/G2-M
Downstream analysis with the High Quality (●) OSNs

Supplementary Figure S3, related to Figure 3 – Quality control (QC) of the single-cell RNA-seq data. (A) Capture rate in the C1 IFC (10-17 µm, Fluidigm): 30 wells captured more than one cell and/or debris, 58 captured single cells, and 8 were empty. The 58 captured single OSNs were subjected to additional quality control (QC) steps; the total fragments obtained for each cell show a bimodal distribution, with 28 samples having low-yield (black) and 30 samples a significantly higher yield (green). (B) Further mapping statistics were analyzed. The total fragments are plotted against: % unmapped fragments, % multi-mapped fragments, % fragments mapped to ERCC spike-ins, % fragments mapped to mt-Rnr2 (the most abundant mitochondrial gene in the samples), and the sum of the 7 most abundant mitochondrial genes. Finally we performed Principal Component Analysis (PCA) of all the above parameters combined; the amount of variance explained by each component is indicated in parentheses. Based on these, it is evident that the low-yield samples are of poor quality. These samples were thus not analyzed further. (C) Projection of the single-cells onto the cell cycle PCA that differentiates between cell cycle stages (See Supplementary Fig. S2B, and Methods for details), shows that all the samples are in the G1 stage, as evidenced by their negative PC1 values.

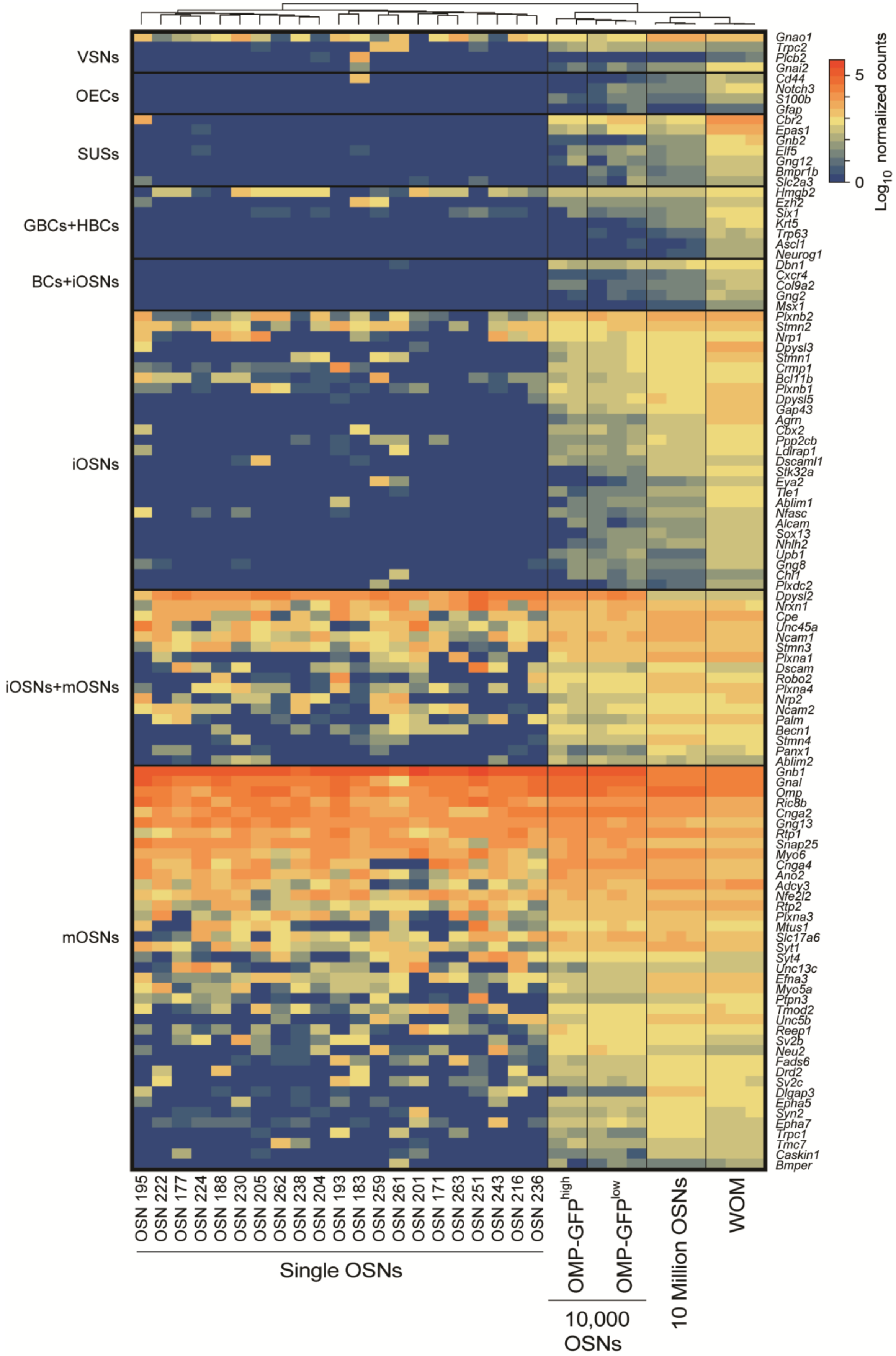
VSNs
OECs
SUSs
GBCs+HBCs
BCs+iOSNs
iOSNs
iOSNs+mOSNs
mOSNs
Gnao1
Trpc2
Plcb2
Gnai2
Cd44
Notch3
S100b
Gfap
Cbr2
Epas1
Gnb2
Elf5
Gng12
Bmpr1b
Slc2a3
Hmgb2
Ezh2
Six1
Krt5
Trp63
Ascl1
Neurog1
Dbn1
Cxcr4
Col9a2
Gng2
Msx1
Plxnb2
Stmn2
Nrp1
Dpysl3
Stmn1
Crmp1
Bcl11b
Plxnb1
Dpysl5
Gap43
Agrn
Cbx2
Ppp2cb
Ldlrap1
Dscaml1
Stk32a
Eya2
Tle1
Ablim1
Nfasc
Alcam
Sox13
Nhlh2
Upb1
Gng8
Chl1
Plxdc2
Dpysl2
Nrxn1
Cpe
Unc45a
Ncam1
Stmn3
Plxna1
Dscam
Robo2
Plxna4
Nrp2
Ncam2
Palm
Becn1
Stmn4
Panx1
Ablim2
Gnb1
Gnal
Omp
Ric8b
Cnga2
Gng13
Rtp1
Snap25
Myo6
Cnga4
Ano2
Adcy3
Nfe2l2
Rtp2
Plxna3
Mtus1
Slc17a6
Syt1
Syt4
Unc13c
Efna3
Myo5a
Ptpn3
Tmod2
Unc5b
Reep1
Sv2b
Neu2
Fads6
Drd2
Sv2c
Dlgap3
Epha5
Syn2
Epha7
Trpc1
Tmc7
Caskin1
Bmper
5
0
Log10 normalized counts
OSN 195
OSN 222
OSN 177
OSN 224
OSN 188
OSN 230
OSN 205
OSN 262
OSN 238
OSN 204
OSN 193
OSN 183
OSN 259
OSN 261
OSN 201
OSN 171
OSN 263
OSN 251
OSN 243
OSN 216
OSN 236
Single OSNs
OMP-GFPhigh
OMP-GFPlow
10,000
OSNs
10 Million OSNs
WOM

Supplementary Figure S4, related to Figure 4 – Captured single cells express marker genes of mature OSNs. Heatmap of the expression values of a comprehensive list of markers for various populations of cells within the main olfactory and vomeronasal mucosae of the mouse [19,23-25], for all OSN samples analyzed. mOSNs: mature OSNs, iOSNs: immature OSNs, GBCs: globose basal cells, HBCs: horizontal basal cells, BCs: globose or horizontal basal cells; SUSs: sustentacular cells, OECs: olfactory ensheating cells; VSNs: vomeronasal sensory neurons.

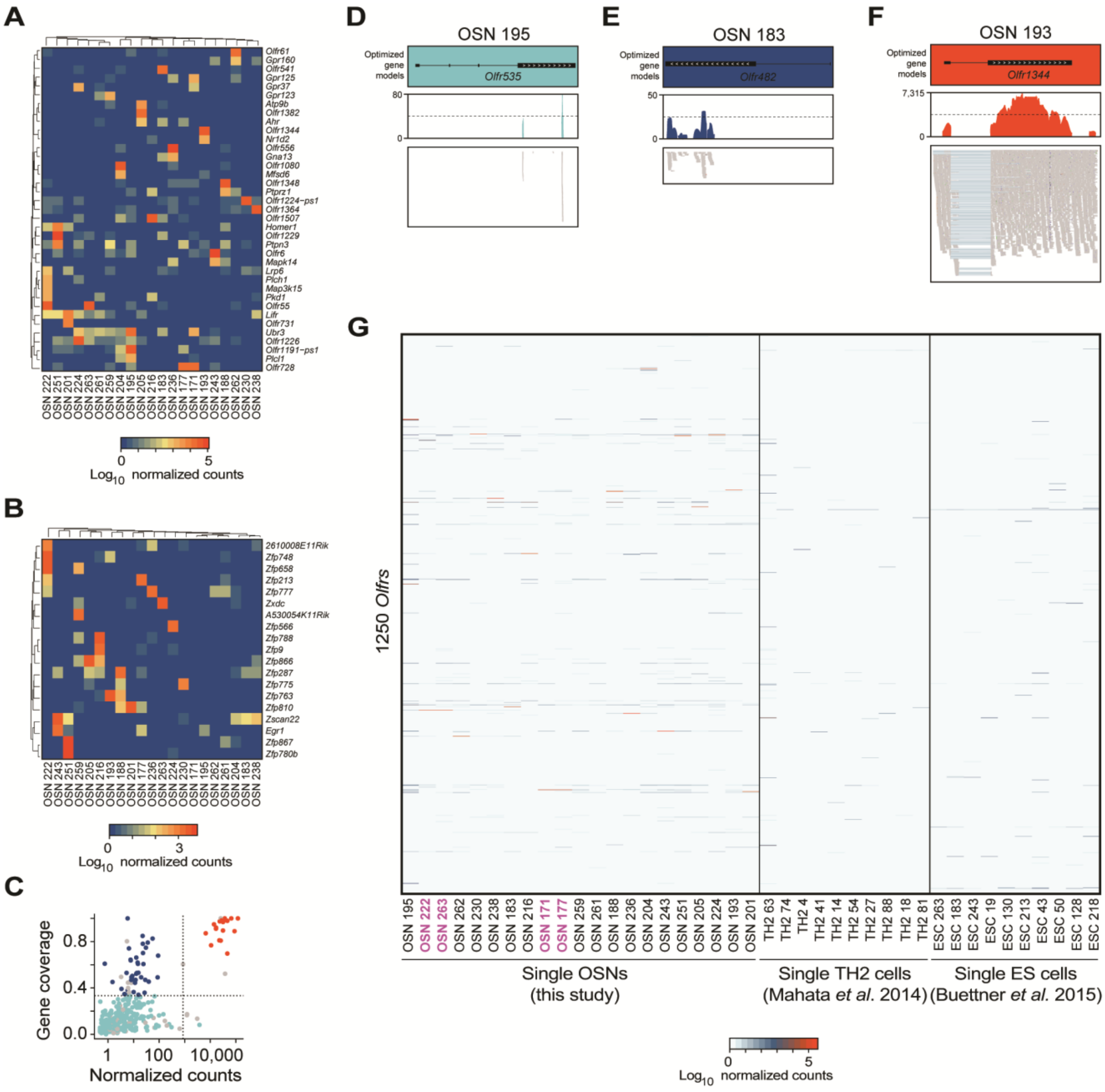
A
Olfr61
Gpr160
Olfr541
Gpr125
Gpr37
Gpr123
Atp9b
Olfr1382
Ahr
Olfr1344
Nr1d2
Olfr556
Gna13
Olfr1080
Mfsd6
Olfr1348
Ptprz1
Olfr1224-ps1
Olfr1364
Olfr1507
Homer1
Olfr1229
Ptpn3
Olfr6
Mapk14
Lrp6
Plch1
Map3k15
Pkd1
Olfr55
Lifr
Olfr731
Ubr3
Olfr1226
Olfr1191-ps1
Plcl1
Olfr728
OSN 222
OSN 251
OSN 201
OSN 224
OSN 263
OSN 261
OSN 259
OSN 204
OSN 195
OSN 205
OSN 216
OSN 183
OSN 236
OSN 177
OSN 171
OSN 193
OSN 243
OSN 188
OSN 262
OSN 230
OSN 238
0
5
Log10 normalized counts
B
2610008E11Rik
Zfp748
Zfp658
Zfp213
Zfp777
Zxdc
A530054K11Rik
Zfp566
Zfp788
Zfp9
Zfp866
Zfp287
Zfp775
Zfp763
Zfp810
Zscan22
Egr1
Zfp867
Zfp780b
OSN 222
OSN 243
OSN 251
OSN 259
OSN 205
OSN 216
OSN 193
OSN 188
OSN 201
OSN 177
OSN 236
OSN 263
OSN 224
OSN 230
OSN 171
OSN 195
OSN 262
OSN 261
OSN 204
OSN 183
OSN 238
0
3
Log10 normalized counts
C
Gene coverage
0.8
0.4
0.0
1
100
10,000
Normalized counts
D
OSN 195
Optimized gene models
Olfr535
80
0
E
OSN 183
Optimized gene models
Olfr482
50
0
F
OSN 193
Optimized gene models
Olfr1344
7,315
0
G
1250 Olfrs
OSN 195
OSN 222
OSN 263
OSN 262
OSN 230
OSN 238
OSN 183
OSN 216
OSN 171
OSN 177
OSN 259
OSN 261
OSN 188
OSN 236
OSN 204
OSN 243
OSN 251
OSN 205
OSN 224
OSN 193
OSN 201
TH2 63
TH2 74
TH2 4
TH2 41
TH2 14
TH2 54
TH2 27
TH2 88
TH2 18
TH2 81
ESC 263
ESC 183
ESC 243
ESC 19
ESC 130
ESC 213
ESC 43
ESC 50
ESC 128
ESC 218
Single OSNs (this study)
Single TH2 cells (Mahata et al. 2014)
Single ES cells (Buettner et al. 2015)
0
5
Log10 normalized counts

Supplementary Figure S5, related to Figure 5 – OR expression in single-cell RNA-seq. (A) Heatmap of the expression levels of genes with a coefficient of variation (CV) greater than 4 that are in GO categories related to GPCR signaling. (B) Heatmap of the expression values of a group of zinc finger protein genes with a CV > 4. (C) Plot of the normalized counts for OR genes versus the proportion of the gene covered by the sequencing data. Each dot represents one OR gene with at least one mapped fragment in a specific cell. The horizontal dotted line indicates a coverage value of a third of the gene; genes below it are in light blue and represent the vast majority (> 85%). The rest of the ORs segregate into two populations of lowly (dark blue) and highly expressed (red) genes. The vertical dotted line is the threshold that separates them (from Fig. 5B). Grey dots are OR genes annotated as pseudogenes. (D) Example of an OR gene with low read coverage (light-blue dots in C). The sequencing fragments are stacked in very restricted regions of the gene, which is not consistent with *bona fide* transcription. (E) Example of an OR gene that has higher read coverage (~50%), but also expressed at a very low level (dark-blue dots in C). Very few fragments are mapped to this OR. (F) Example of an OR gene that has high expression across the entire length of the transcript (red dots in C). In (D-F) The top panels represent gene models from [14]; the middle panels represent the fragment coverage, the y-axis in counts varies in scale; the bottom panels show the actual reads mapped to each gene; blue lines join portions of a read that spans exon junctions. (G) Heatmap of the normalized expression levels of 1,250 OR genes in single OSNs, with representative, examples of 10 (out of 96) single Th2 cells [26] and 10 (out of 288) single ES cells [3]. For the majority of single OSNs, a highly expressed OR gene is apparent (orange) with a varying number of lowly expressed OR genes (dark blue); there is no consistent pattern in the lowly expressed OR genes, even between those cells that express the same abundant OR gene (highlighted in pink in the x-axis). TH2 and ES cells also express variable numbers of different OR genes, but always at low levels.

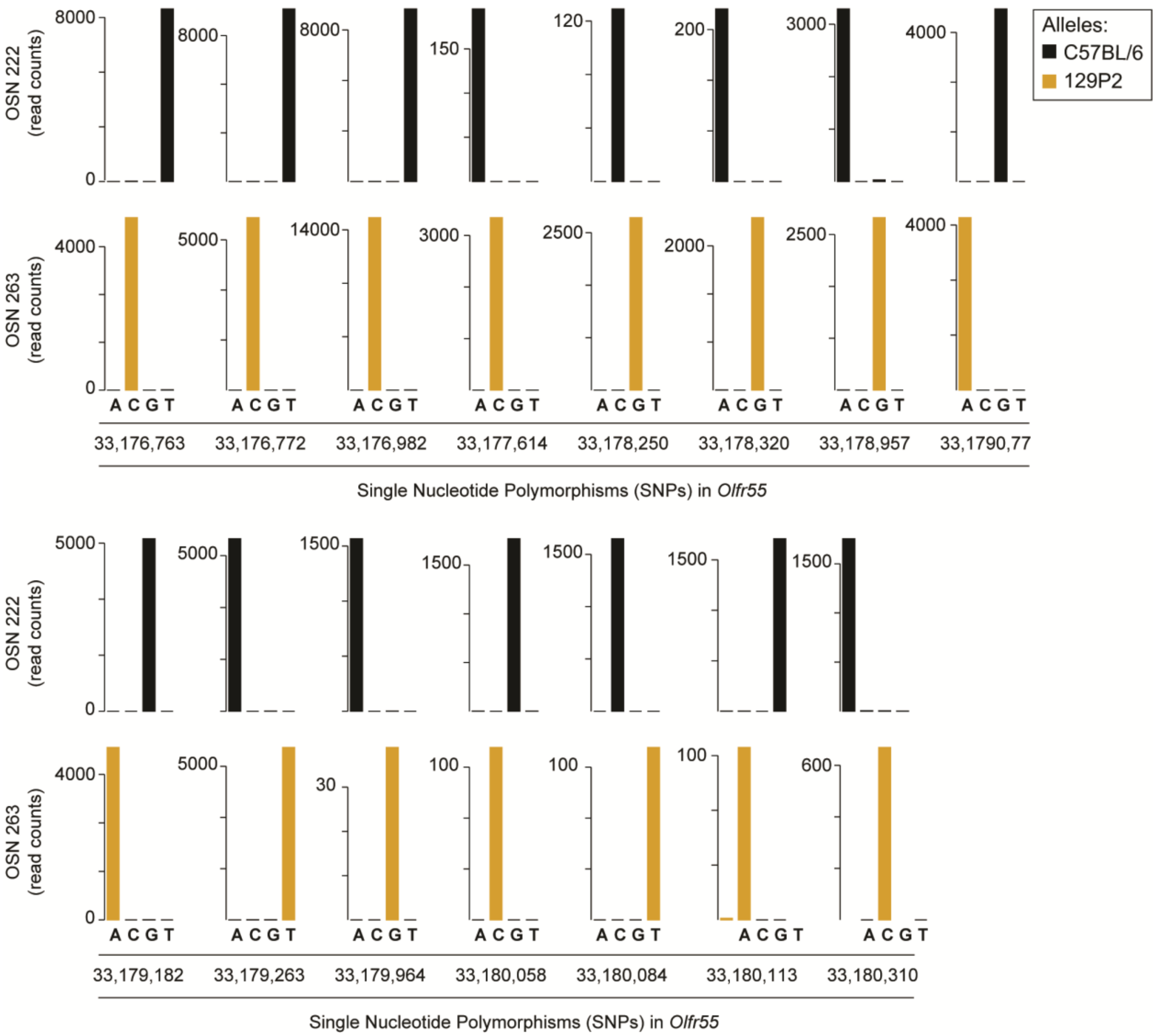

Alleles:
C57BL/6
129P2
OSN 222 (read counts)
OSN 263 (read counts)
A C G T
33,176,763
33,176,772
33,176,982
33,177,614
33,178,250
33,178,320
33,178,957
33,1790,77
Single Nucleotide Polymorphisms (SNPs) in Olfr55
33,179,182
33,179,263
33,179,964
33,180,058
33,180,084
33,180,113
33,180,310
Single Nucleotide Polymorphisms (SNPs) in Olfr55

Supplementary Figure S6, related to Figure 6 – OR gene expression is monoallelic. For each of the 15 informative SNPs in *Olfr55*, the number of reads containing each nucleotide, A, C, G or T, is plotted for OSN 222 (top) and OSN 263 (bottom). Black and gold indicate the nucleotide that corresponds to the C57BL/6 and 129P2 alleles respectively. For OSN 222 all SNP positions bear the B6 allele, while for OSN 263 all SNPs are of the 129P2 allele.

A

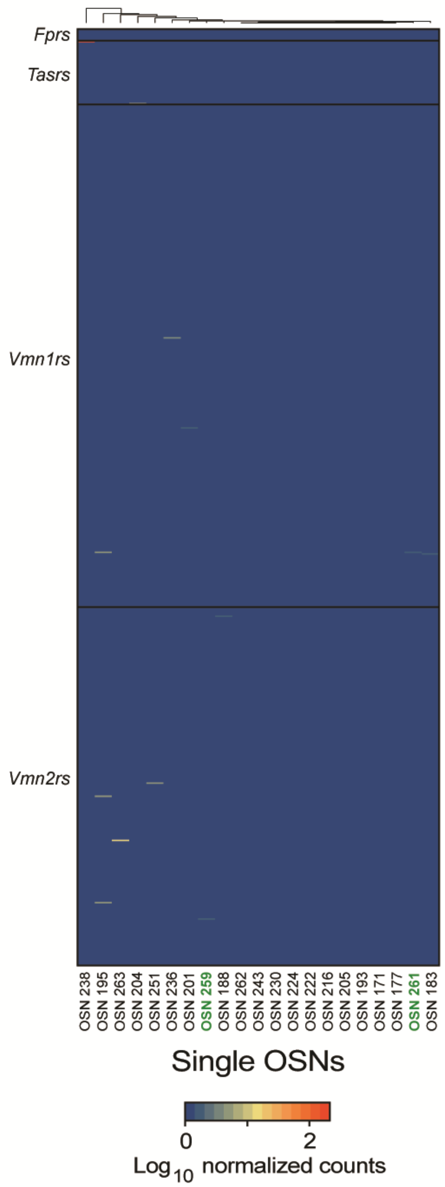
Fprs
Tasrs
Vmn1rs
Vmn2rs
OSN 238
OSN 195
OSN 263
OSN 204
OSN 251
OSN 236
OSN 201
OSN 259
OSN 188
OSN 262
OSN 243
OSN 230
OSN 224
OSN 222
OSN 216
OSN 205
OSN 193
OSN 171
OSN 177
OSN 261
OSN 183
Single OSNs
0
2
Log10 normalized counts

B

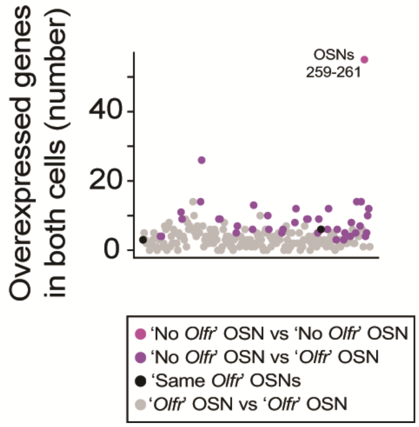
Overexpressed genes in both cells (number)
0
20
40
OSNs 259-261
'No Olfr' OSN vs 'No Olfr' OSN
'No Olfr' OSN vs 'Olfr' OSN
'Same Olfr' OSNs
'Olfr' OSN vs 'Olfr' OSN

Supplementary Figure S7, related to Figure 7 – OSNs lacking abundant OR gene expression are a molecularly distinct type of neuron. (A) Heatmap of the normalized expression levels for formyl peptide (*Fprs*), taste (*Tasrs*) and vomeronasal type 1 (*Vmn1rs*) and type 2 (*Vmn2rs*) receptor genes. OSNs lacking OR gene expression are indicated (green). (B) Each dot represents a different combination of two cells tested against the other 19 in a transcriptome-wide differential expression (DE) analysis. The number of DE genes that are significantly upregulated in both cells is plotted on the y-axis. The two cells lacking abundant OR gene expression, OSN 259 and OSN 261 in pink, have 55 differentially upregulated genes. Other combinations containing one of these cells are in purple, and the remainder in grey. All other combinations show considerably lower numbers of DE genes. Further highlighted in black are the two pairs of cells that express the same OR gene; these are no more similar to each other as they are to any other OR-expressing cell.

Supplementary Data S1, related to Figure 1 – The transcriptomes of the whole olfactory mucosa and pools of 10 million OSNs. Excel workbook containing the mapping statistics of the RNA-seq data for each sample, along with the accession numbers for the raw data; the normalized counts for the whole transcriptome; the differential expression analysis results between the WOM and the 10 million pooled OSNs; the GO categories significantly over-represented in the genes specifically expressed in the OSNs (fold-change > 3).

Supplementary Data S2, related to Figure 2 – The transcriptomes of OMP-GFP^{low} and OMP-GFP^{high} OSNs. Excel workbook containing the mapping statistics of the RNA-seq data for each sample, along with the accession numbers for the raw data; the normalized counts for the whole transcriptome; the differential expression analysis results between the OMP-GFP^{low} and OMP-GFP^{high} samples; the GO categories significantly over-represented in the differentially expressed genes.

Supplementary Data S3, related to Figure 3 – The transcriptomes of single OSNs. Excel workbook containing the mapping statistics of the RNA-seq data for each cell, along with the accession numbers for the raw data; the normalized counts for the whole transcriptome for the 21 cells analyzed in this manuscript, along with 9 additional cells that were excluded in the last QC step (see Methods); the differential expression analysis results between the two cells lacking abundant OR expression and the other 19; the GO categories and PFAM domains significantly over-represented in the genes with a coefficient of variation greater than 4; the normalized counts of all genes with a coefficient of variation greater than 4.

Supplementary methods

Overrepresentation analysis of functional terms. To find functional terms enriched in the lists of differentially expressed genes, GeneTrail was used (http://genetrail.bioinf.uni-sb.de/) with 'Over- / Under-representation Analysis'. The background provided were all those genes tested for differential expression (those with an adjusted p-value different to NA). Detailed results can be found in Supplementary Data S1-3.

Cell cycle stage allocation. In order to assess the cell-cycle stage of a given bulk or single-cell RNA-seq dataset (test dataset), we used a recently published single-cell RNA-seq dataset (training dataset) [1] composed of stem cells that had been sorted by cell-cycle stage prior to the sequencing of their transcriptome. Both the training and the testing datasets were first normalized for sequencing depth. The two datasets were then pooled together and a quantile normalization was carried out on the genes that are annotated as part of the "cell cycle" in the Gene Ontology database (GO:0007049) [2]. Genes that had no counts in all samples were excluded. A Principal Component Analysis (PCA) on the training dataset shows that the first principal component (PC1) approximately tracks the cell-cycle phase (see Fig. S2B and [3]). In particular, all G1 cells (except a few outliers) have a negative value on PC1, while the PC1 value of S and G2-M cells is positive. Therefore, we reasoned that by projecting a test dataset onto this PCA we would be able to distinguish G1 from S/G2M cells by the sign of PC1. As a positive control, we applied this to published single-cell and bulk RNA-seq datasets from terminally differentiated mouse cells collected from the liver [4] and the brain [5], which are correctly predicted to be in G1 phase (see Supplementary Fig. S2C). On the other hand, blastomeres collected from 2-cell stage mouse embryos [4] that are most likely to be either in S or in G2-M phase [6], consistently have a positive value for the PC1 (see Supplementary Fig. S2C). We then applied this same methodology to our bulk and single-cell RNA-seq data as test datasets, and

found that all samples have a negative PC1 (Fig. 2G and Supplementary Fig. S3), suggesting that single OSNs are in G1 phase and that bulk samples include mostly cells in G1.

Quality control of single-cell data. The quality of the RNA-seq data produced from a single cell is greatly influenced by the amount and integrity of the starting material. Including cells with poor quality data in normalization steps can be detrimental to the downstream analyses of cells with good quality data. Therefore it is imperative that such cells be identified and excluded at an early stage [7]. For the purpose of this study, we were interested only in single cells, so C1 Single-Cell Auto Prep IFC wells that contained multiple cells or debris were not analyzed further (30 samples), nor were samples from empty wells (8). From the remaining 58, the distribution of total fragments obtained per sample was clearly bimodal (Supplementary Fig. S3A). Deconvolution into two normal-like distributions revealed 28 cells with low yield (mean of 1.7 million) while the remaining 30 were sequenced at significant higher levels (mean of 4.4 million; t-test, $P < 2.2e-16$). To determine if the lower yield was a result of sequencing poor quality libraries, we analyzed mapping statistics (Supplementary Fig. S3B). The low-yield group of samples had a much higher percentage of unmapped fragments (31.97% on average versus 10.21% in the high-yield group; t-test P=5.739e-08) as well as multi-mapped fragments (20.27% versus 5.49%; t-test, P= 4.018e-08). The proportion of fragments that mapped to ERCC spike-ins was over 20 times higher in the low- versus high-yield groups (t-test, P=5.576e-05). Furthermore, most of the uniquely mapped fragments from the low yield samples aligned to mitochondrial genes (on average 60.53%, compared to 4.77% in the high-yield samples; t-test, P=6.327e-12). Together these quality controls suggest that the starting material for the samples with low yield was of poor quality; we therefore focused our subsequent analysis on the 30 high-yield samples. We next compared the OR gene expression profiles of these samples as a function of their capture location on the C1 Single-Cell Auto Prep IFC chip and the Nextera XT library preparation plate. Ten cells (OSN 157, 178, 185, 191, 207, 214, 218, 223, 255 and 263)

had evidence of two highly expressed OR genes. OSN 263 had high counts for *Olfr55* and *Olfr239*, two adjacent OR genes that are 99% identical. Closer inspection of the sequencing data revealed that the fragments assigned to *Olfr239* were in fact mismapped, such that a BLAST alignment maps them back to *Olfr55*. We therefore set the counts of *Olfr239* to zero. For the remaining nine cells, in four cases (OSN 178, 191, 223 and 255) we found that one OR gene was expressed in another sample located in the immediately adjacent well, suggesting evidence of carry-over. The other five cells (OSN 185 (*Olfr1348* and *Olfr571*), 207 (*Olfr1463*, *Olfr476* and *Olfr733*), 214 (*Olfr1258* and *Olfr716*), 218 (*Olfr1419* and *Olfr6*) and 257 (*Olfr46* and *Olfr873*)) did not share an OR gene with a sample in an adjacent well. We independently reassessed these nine cells through all previous quality control criteria and could not distinguish the four cells with evidence of carry-over from the five cells without such evidence. A recent report found that up to 20% of cells captured on a C1 microfluidic system contain two cells that are not visible in the microscopy images [8]. Thus, to take a conservative approach and to reduce the possibility of including samples containing a second, visually obscured cell or contaminating debris in our subsequent analysis, we elected to exclude all 9 from this study. This procedure resulted in the final dataset of 21 samples of single OSNs that were further analyzed and are presented in this paper.

Coverage of OR genes. To obtain the proportion of the OR gene models covered by the mapped sequencing fragments, the BEDtools 2.16.2 [9] program *coverageBed* was used against a BED file containing the merged exonic regions for all isoforms of each OR gene (obtained with *mergeBed*). The output was then analyzed in R to count all positions with at least one mapped fragment to them.

Sequencing data visualization. Sequencing data was visualized using the Integrative Genomics Viewer (IGV) [10,11] and the UCSC Genome Browser. For the latter, BAM files were converted to bigWig using BEDtools 2.16.2 [9] *genomeCoverageBed* program and the *wigToBigWig* utility (http://hgdownload.cse.ucsc.edu/admin/exe/).

Phylogenetic reconstruction of the OR gene repertoire. A total of 1,098 OR genes with full-length open reading frames (ORFs) were extracted from the Ensembl mouse genome database, version 72. The longest ORF annotated for each gene was translated to generate amino acid sequence, and these were aligned using Clustal Omega with default parameters. Phylogenetic reconstruction was conducted in MEGA4 using the Minimum Evolution method [12]. The neighbor-joining algorithm was used to generate an initial tree [13]. All positions containing alignment gaps and missing data were eliminated only in pairwise sequence comparisons (using the pairwise deletion option). There were a total of 608 positions in the final dataset.

Fluorescent in situ hybridization (ISH). For single-color ISH, tissue preparation, hybridization and washing steps were performed as described [14], in cryosections of the MOE of 8-week-old male C57BL/6J mice. The ribopobes for *Gucy1b2* and *Sln* were prepared as previously described [15], using the following primers: Gucy1b2_fw, *GCTGGACACCATGTACGGAT*; Gucy1b2_rv, *TCCCACGTCTCCTCTCCAAA*. Sln_fw, *CCAATACTGAGGGGCCATGC*; Sln_Rv, *TCCTACATTCTTCCTTGGGGC*. Riboprobes were labeled with DIG or FLU, and visualized using sheep anti-DIG-POD or anti-FLU-POD (Roche) and the direct TSA-FITC Plus Kit (Perkin Elmer). Images were collected with a Zeiss Axiovert 200M microscope.

For two-color ISH, tissue preparation, hybridization and washing steps were performed as described [16], in cryosections of the MOE of 3-week-old male or female C57BL/6J mice. Riboprobes were prepared for *Sln* (nucleotides 12-521 from GenBank accession number NM_025540.2), *Emx1* (nucleotides 426-1136 from GenBank accession number NM_010121.2),

Sncg (nucleotides 1-685 from GenBank accession number NM_01143.3). *Gucy1b2* was from reference [17], and *Trpc2* was from reference [18]. *Gucy1b2* riboprobe was labeled with DNP and detected with rabbit anti-DNP antibody (Life Technologies) and goat anti-rabbit IgG Alexa488 (Life Technologies), *Sln, Emx1, Trpc2* and *Sncg* riboprobes were labeled with DIG and detected with sheep anti-DIG-AP (Roche) and HNPP/Fast Red TR detection system (Roche). Images were collected with a Zeiss LSM 710 confocal microscope.

Supplementary references

1 Sasagawa, Y. *et al.* Quartz-Seq: a highly reproducible and sensitive single-cell RNA sequencing method, reveals non-genetic gene-expression heterogeneity. *Genome Biol* **14**, R31 (2013).

2 Gene Ontology, C. Gene Ontology Consortium: going forward. *Nucleic Acids Res* **43**, D1049-1056 (2015).

3 Buettner, F. *et al.* Computational analysis of cell-to-cell heterogeneity in single-cell RNA-sequencing data reveals hidden subpopulations of cells. *Nat Biotechnol* **33**, 155-160 (2015).

4 Deng, Q., Ramskold, D., Reinius, B. & Sandberg, R. Single-cell RNA-seq reveals dynamic, random monoallelic gene expression in mammalian cells. *Science* **343**, 193-196 (2014).

5 Schmitt, B. M. *et al.* High-resolution mapping of transcriptional dynamics across tissue development reveals a stable mRNA-tRNA interface. *Genome Res* **24**, 1797-1807 (2014).

6 Ciemerych, M. A. & Sicinski, P. Cell cycle in mouse development. *Oncogene* **24**, 2877-2898 (2005).

7 Stegle, O., Teichmann, S. A. & Marioni, J. C. Computational and analytical challenges in single-cell transcriptomics. *Nat Rev Genet* **16**, 133-145 (2015).

8 Macosko, E. Z. *et al.* Highly parallel genome-wide expression profiling of individual cells using nanoliter droplets. *Cell* **161**, 1202-1214 (2015).

9 Quinlan, A. R. & Hall, I. M. BEDTools: a flexible suite of utilities for comparing genomic features. *Bioinformatics* **26**, 841-842 (2010).

10 Thorvaldsdottir, H., Robinson, J. T. & Mesirov, J. P. Integrative Genomics Viewer (IGV): high-performance genomics data visualization and exploration. *Brief Bioinform* **14**, 178-192 (2013).

11 Robinson, J. T. *et al.* Integrative genomics viewer. *Nat Biotechnol* **29**, 24-26 (2011).

12 Tamura, K., Dudley, J., Nei, M. & Kumar, S. MEGA4: Molecular Evolutionary Genetics Analysis (MEGA) software version 4.0. *Mol Biol Evol* **24**, 1596-1599 (2007).

13 Saitou, N. & Nei, M. The neighbor-joining method: a new method for reconstructing phylogenetic trees. *Mol Biol Evol* **4**, 406-425 (1987).

14 Ibarra-Soria, X., Levitin, M. O., Saraiva, L. R. & Logan, D. W. The olfactory transcriptomes of mice. *PLoS Genetics* **10(9)**, e1004593 (2014).

15 Saraiva, L. R. & Korsching, S. I. A novel olfactory receptor gene family in teleost fish. *Genome Res* **17**, 1448-1457 (2007).

16 Ishii, T., Omura, M. & Mombaerts, P. Protocols for two- and three-color fluorescent RNA in situ hybridization of the main and accessory olfactory epithelia in mouse. *J Neurocytol* **33**, 657-669 (2004).

17 Omura, M. & Mombaerts, P. Trpc2-expressing sensory neurons in the mouse main olfactory epithelium of type B express the soluble guanylate cyclase Gucy1b2. *Mol Cell Neurosci* **65**, 114-124 (2015).

18 Omura, M. & Mombaerts, P. Trpc2-expressing sensory neurons in the main olfactory epithelium of the mouse. *Cell Rep* **8**, 583-595 (2014).

19 Sammeta, N., Yu, T. T., Bose, S. C. & McClintock, T. S. Mouse olfactory sensory neurons express 10,000 genes. *J Comp Neurol* **502**, 1138-1156 (2007).

20 Clowney, E. J. *et al.* Nuclear aggregation of olfactory receptor genes governs their monogenic expression. *Cell* **151**, 724-737 (2012).

21 Dalton, R. P., Lyons, D. B. & Lomvardas, S. Co-opting the unfolded protein response to elicit olfactory receptor feedback. *Cell* **155**, 321-332 (2013).

22 Mori, K. & Sakano, H. How is the olfactory map formed and interpreted in the mammalian brain? *Annu Rev Neurosci* **34**, 467-499 (2011).

23 Higginson, J. R. & Barnett, S. C. The culture of olfactory ensheathing cells (OECs)--a distinct glial cell type. *Exp Neurol* **229**, 2-9 (2011).

24 Yu, T. T. *et al.* Differentially expressed transcripts from phenotypically identified olfactory sensory neurons. *J Comp Neurol* **483**, 251-262 (2005).

25 Nickell, M. D., Breheny, P., Stromberg, A. J. & McClintock, T. S. Genomics of mature and immature olfactory sensory neurons. *J Comp Neurol* **520**, 2608-2629 (2012).

26 Mahata, B. *et al.* Single-cell RNA sequencing reveals T helper cells synthesizing steroids de novo to contribute to immune homeostasis. *Cell Rep* **7**, 1130-1142 (2014).
